# Effects of exercise on circulating tumor cells among patients with resected stage I-III colon cancer

**DOI:** 10.1371/journal.pone.0204875

**Published:** 2018-10-17

**Authors:** Justin C. Brown, Andrew D. Rhim, Sara L. Manning, Luke Brennan, Alexandra I. Mansour, Anil K. Rustgi, Nevena Damjanov, Andrea B. Troxel, Michael R. Rickels, Bonnie Ky, Babette S. Zemel, Kerry S. Courneya, Kathryn H. Schmitz

**Affiliations:** 1 Dana-Farber Cancer Institute, Boston, MA, United States of America; 2 MD Anderson Cancer Center, Houston, TX, United States of America; 3 University of Michigan Medical School, Ann Arbor, MI, United States of America; 4 University of Pennsylvania, Philadelphia, PA, United States of America; 5 New York University, New York, NY, United States of America; 6 Childrens Hospital of Philadelphia, Philadelphia, PA, United States of America; 7 University of Alberta, Edmonton, Alberta, Canada; 8 Penn State College of Medicine, Hershey, PA, United States of America; George Washington University Milken Institute of Public Health, UNITED STATES

## Abstract

**Background:**

Physical activity is associated with a lower risk of disease recurrence among colon cancer patients. Circulating tumor cells (CTC) are prognostic of disease recurrence among stage I-III colon cancer patients. The pathways through which physical activity may alter disease outcomes are unknown, but may be mediated by changes in CTCs.

**Methods:**

Participants included 23 stage I-III colon cancer patients randomized into one of three groups: usual-care control, 150 min∙wk^-1^ of aerobic exercise (low-dose), and 300 min∙wk^-1^ of aerobic exercise (high-dose) for six months. CTCs from venous blood were quantified in a blinded fashion using an established microfluidic antibody-mediated capture device. Poisson regression models estimated the logarithmic counts of CTCs.

**Results:**

At baseline, 78% (18/23) of patients had ≥1 CTC. At baseline, older age (−0.12±0.06; *P* = 0.04), lymphovascular invasion (0.63±0.25; *P* = 0.012), moderate/poor histology (1.09±0.34; *P* = 0.001), body mass index (0.07±0.02; *P* = 0.001), visceral adipose tissue (0.08±0.04; *P* = 0.036), insulin (0.06±0.02; *P* = 0.011), sICAM-1 (0.04±0.02; *P* = 0.037), and sVCAM-1 (0.06±0.03; *P* = 0.045) were associated with CTCs. Over six months, significant decreases in CTCs were observed in the low-dose (−1.34±0.34; *P*<0.001) and high-dose (−1.18±0.40; *P* = 0.004) exercise groups, whereas no significant change was observed in the control group (−0.59±0.56; *P* = 0.292). Over six months, reductions in body mass index (−0.07±0.02; *P* = 0.007), insulin (−0.08±0.03; *P* = 0.014), and sICAM-1 (−0.07±0.03; *P* = 0.005) were associated with reductions in CTCs. The main limitations of this proof-of-concept study are the small sample size, heterogenous population, and per-protocol statistical analysis.

**Conclusion:**

Exercise may reduce CTCs among stage I-III colon cancer patients. Changes in host factors correlated with changes in CTCs. Exercise may have a direct effect on CTCs and indirect effects through alterations in host factors. This hypothesis-generating observation derived from a small pilot study warrants further investigation and replication.

## Introduction

The development of metastases from colon cancer are hypothesized to result from tumor cells entering the circulation, migrating to distant organs, extravasting, multiplying, and eventually manifesting as clinically-detectable lesions [1]. The early detection and characterization of circulating tumor cells (CTCs) are important to monitor and prevent the development of metastases [2]. CTCs predict disease recurrence and mortality among patients with stage I-III colon cancer [3–6]. For example, among stage III colon cancer patients, the presence of ≥1 CTC(s) after completing post-operative chemotherapy is independently associated with a six-fold increase in the risk of disease recurrence [6].

Exercise after a diagnosis of stage I-III colon cancer may lower the risk of disease recurrence and mortality [7, 8]. However, the biologic or biobehavioral mechanisms through which exercise may improve disease outcomes are not known. Computational models of fluid shear stress demonstrate that shear flow characteristics may affect CTC viability and alter intracellular characteristics of CTCs [9], such that higher forces and longer durations of shear stress exposure may retard growth rates of CTCs and attenuate metastatic potential [10, 11]. Exercise induces substantial increases in vascular shear stress [12, 13], which may alter CTCs in patients with colon cancer. This direct effect of exercise on CTCs may explain, in part, the biological mechanism through which exercise reduces disease recurrence in patients with colon cancer.

In addition, patient or host characteristics related to energy balance [14], such as obesity and hyperinsulinemia, may create an environment that is plentiful in growth factors and impaired immune surveillance necessary to promote the survival and proliferation of CTCs [15], perhaps explaining why states of obesity and hyperinsulinemia are associated with disease recurrence and mortality [16–18]. Exercise reduces visceral obesity and insulin among patients with colon cancer [19, 20]. Exercise may therefore also have indirect effects on CTCs by altering the host tumor microenvironment.

We explored these novel ideas by enumerating CTCs in the setting of a pilot randomized exercise trial among patients with resected stage I-III colon cancer [21]. Given the hypothesis-generating nature of this study, we aimed to characterize: 1) the proportion of colon cancer patients with enumerable CTCs prior to exercise training; 2) demographic, clinical, and host factors that correlated with CTCs prior to exercise training; 3) changes in CTCs after exercise training; and 4) the relationship between changes in host factors (i.e., obesity, insulin) with changes in CTCs.

## Materials and methods

### Participants

Detailed methods of this randomized clinical trial have been published [21]. Patients were eligible for participation if they were diagnosed with histologically-proven stage I-III colon cancer; completed surgical resection and adjuvant chemotherapy within 36 months of entering the study; self-reported participating in <150 min∙wk^-1^ of moderate or vigorous intensity physical activity [22]; were of age ≥18 years; provided written physician approval; had no additional surgery planned within the six-month intervention period; and had the ability to walk unassisted for six minutes.

### Randomization

Participants were stratified on cancer stage and randomized in equal proportion to one of three groups: low-dose aerobic exercise (150 min∙wk^-1^), high-dose aerobic exercise (300 min∙wk^-1^), or usual care control. This study was approved by the University of Pennsylvania Institutional Review Board and registered on Clinicaltrials.gov as NCT02250053. All participants provided informed consent and approval from their physician prior to participation in any study activities.

### Intervention

Participants randomized to the low-dose or high-dose exercise groups were provided with an in-home treadmill (LifeSpan Fitness, TR1200i, Salt Lake City, UT) and a heart rate monitor (Polar Electro, RS400, Kempele Finland). Exercise intensity was prescribed at 50–70% of the age-predicted maximum heart rate. The low-dose and high-dose groups progressed toward of the goal of 150 or 300 min∙wk^-1^ of exercise, respectively. Detailed methods and results of the exercise intervention are published [21, 23]. Over six months, the low-dose group completed 141 min∙wk^-1^ (95% CI: 122−161; 93% adherence) and the high-dose group completed 247 min·wk^-1^ (95% CI: 226−268; 89% adherence) of exercise. Participants randomized to the usual-care control group were asked to maintain their pre-study levels of physical activity or follow the recommendations provided by their physician.

### Measurements

Baseline and six-month measurements were obtained by trained staff members who were blinded to treatment assignment. Demographic characteristics including age, sex, and race were self-reported. Smoking status was obtained from a standardized questionnaire [24]. Clinical information including cancer stage, T stage (depth of invasion of the primary tumor), N stage (regional lymph node involvement), treatment with chemotherapy, presence of lymphovascular invasion, and histologic tumor differentiation were obtained from the cancer registry, pathology reports, and physician records.

Body mass index (BMI; kg/m^2^) was calculated using standard anthropometric measures [weight (kg) and height (m)], and dual-energy x-ray absorptiometry was used to quantify visceral adipose tissue [21]. All study participants underwent a fasting blood draw at baseline and follow-up. EDTA-preserved plasma was stored at −80°C. Insulin concentration was quantified using a radioimmunoassay (EMD Millipore, Billerica, MA). Insulin-like growth factor 1 (IGF-1), insulin-like growth factor binding protein 3 (IGFBP-3), soluble intercellular adhesion molecule 1 (sICAM-1), and soluble vascular adhesion molecule 1 (sVCAM-1) concentrations were quantified using enzyme-linked immunosorbent assays (DSL, Webster, TX, USA for IGF-1 and IGFBP-3; EMD Millipore, Bellerica, MA, USA for sICAM-1 and sVCAM-1). Baseline and follow-up samples for each participant were assayed simultaneously and in duplicate at the end of the study. Coefficients of variation for all assays were ≤10%.

### Circulating tumor cell enumeration

At baseline and follow-up, a sample of 10 mL whole blood was collected in a tube containing 300 μL of Na_2_EDTA. Samples were processed within 72-hours of collection using a geometrically enhanced differential immunocapture (GEDI) platform [25]. GEDI is a microfluidic device that utilizes obstacles coated with an antibody specific to epithelial cell-adhesion molecule (EpCAM) to capture rare cells within blood. Blood was perfused through the GEDI chip at 1 mL/hr. Captured cells were stained with the nuclear marker DAPI, fluorescently labeled antibodies to the leukocyte marker CD45, and the epithelial cell marker Pdx-1. Using fluorescence microscopy, DAPI+/CD45-/Pdx1+ EpCAM captured cells with intact cellular morphology were counted as CTCs by a technician blinded to randomized treatment assignment. CTC counts are presented per 1 mL of whole blood [26].

### Statistical analysis

Descriptive statistics presented for baseline variables include counts and proportions for categorical variables and means ± standard deviations for continuous variables. Categorical baseline characteristics were compared among the three groups using Fisher’s exact test, and continuous baseline characteristics were compared among the three study groups using the Kruskal-Wallis test. The distribution of CTCs (a count outcome) was positively-skewed (*P*<0.001) with a mean approximately equal to its standard deviation (3.35±3.08). Therefore a series of Poisson regression models were used to estimate CTCs [27]. The Poisson regression model estimates the logarithm of the expected count of CTCs. To evaluate changes in CTCs from baseline to six months in the randomized groups, a random-effects Poisson model was estimated. A negative binomial model was also fit to the data and the results did not differ from those presented herein using Poisson models. We did not adjust our type I error rate for multiplicity. All analyses should be interpreted as exploratory and hypothesis-generating.

## Results

**Fig 1:**
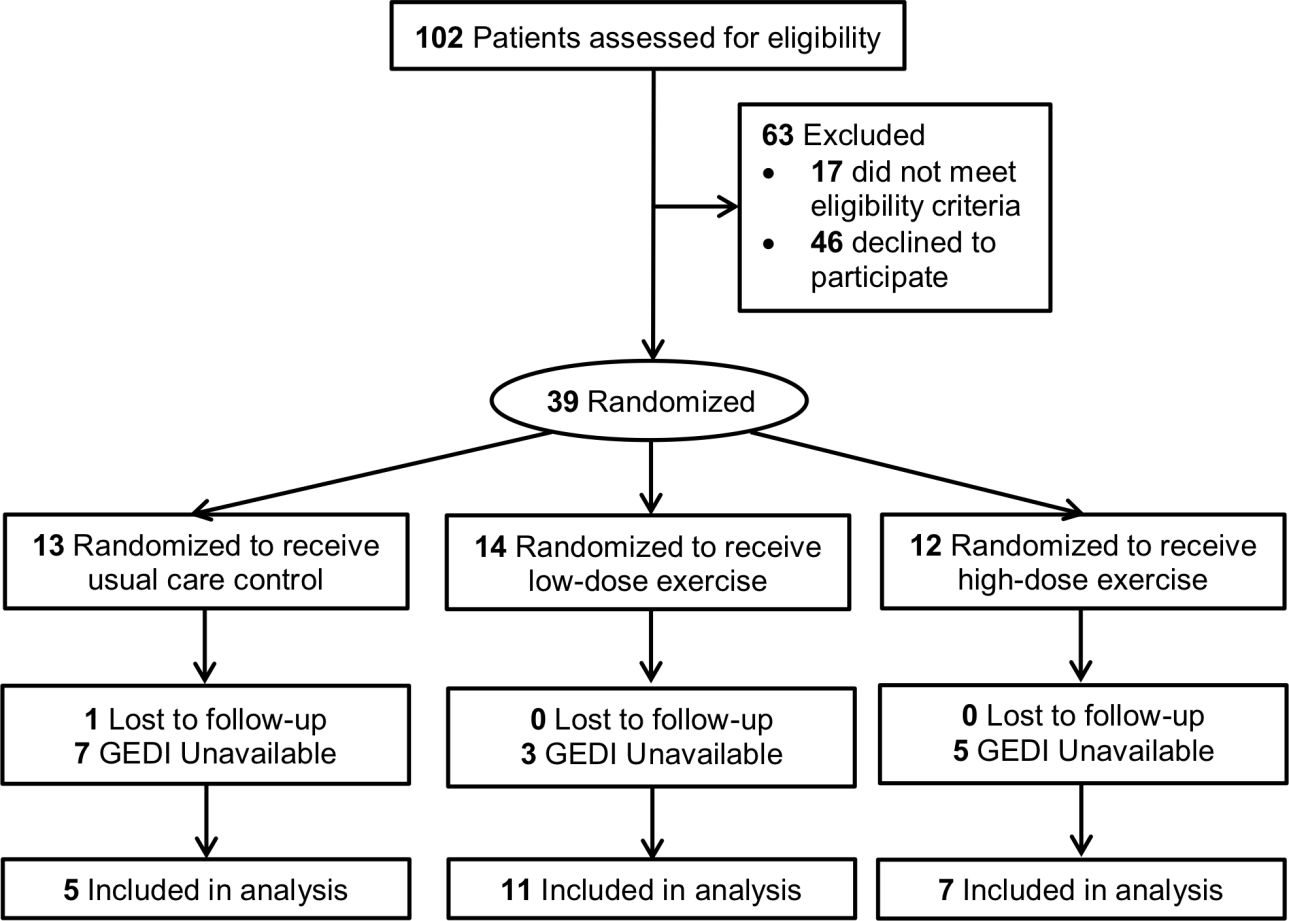
Flow of participants through the study and availability of geometrically enhanced differential immunocapture (GEDI) chips.

The availability of GEDI chips was limited due to our faster than anticipated accrual onto the study protocol. Therefore, 23 of 39 trial participants had both a baseline and six-month CTC enumeration sample available (**Fig 1**). The proportion of participants with available CTC samples were similar across the three randomized groups (*P* = 0.127). The 23 participants with available CTC samples were like the 39 participants in the full study sample (**S1 Table**), with the exception that IGF-1 was lower in participants with CTC enumeration (4.13±0.30 *vs* 4.03±0.23; *P* = 0.032).

The average age of study participants was 55.9±9.3 years. Most patients had stage II (35%) or stage III (52%) colon cancer, and 74% were treated with chemotherapy (all chemotherapy regimens included a fluoropyrimidine and oxaliplatin). Patients completed cancer therapy a median of eight months prior to study enrollment (range: 1−21 months).

At baseline, 78% (18/23) of patients had ≥1 CTC, 65% (15/23) had ≥2 CTCs, and 48% (11/23) had ≥3 CTCs per 1 mL of whole blood. The median number of CTCs was 2 [interquartile range: 1−6] and ranged from 0 to 10. In cross-sectional baseline analyses, several characteristics were associated with CTCs (**Table 1**). Older age was associated with fewer CTCs (−0.12±0.06; *P* = 0.043). Tumors with moderate or poor histologic differentiation (1.09±0.34; *P* = 0.001) and lymphovascular invasion (0.63±0.25; *P* = 0.012) were associated with more CTCs. Higher BMI (0.07±0.02; *P* = 0.001), visceral adipose tissue (0.08±0.04; *P* = 0.036), insulin (0.06±0.02; *P* = 0.011), sICAM-1 (0.04±0.02; *P* = 0.037), and sVCAM-1 (0.06±0.03; *P* = 0.045) were associated with more CTCs.

**Table 1:** Characteristics associated with CTCs at baseline (*n* = 23).

**Characteristic**	**Total Sample Mean or Count (%)**	**CTCs** **(LS Mean ± SE)**	***P***
*Demographic*			
Age, years	55.9±9.3	−0.12±0.06^a^	0.043
Sex			
Male	7 (30%)	0.00—Referent	—
Female	16 (70%)	−0.43±0.23	0.063
Race			
White	19 (83%)	0.00—Referent	—
Black/Other	4 (17%)	−0.60±0.37	0.110
Smoking History			
Never	11 (48%)	0.00—Referent	—
Former/Current	12 (52%)	−0.22±0.23	0.342
*Tumor & Treatment*			
Stage			
I	3 (13%)	0.00—Referent	—
II	8 (35%)	−0.05±0.36	0.896
III	12 (52%)	−0.15±0.34	0.669
T Stage			
T1-T2	3 (13%)	0.00—Referent	—
T3	14 (61%)	−0.33±0.34	0.340
T4	6 (26%)	0.28±0.35	0.435
N Stage			
N0	11 (48%)	0.00—Referent	—
N1-2	12 (52%)	−0.11±0.23	0.620
Chemotherapy	17 (74%)	0.15±0.27	0.588
Time Since Treatment Completion, Months	9.0±6.1	−0.008±0.02	0.662
Lymphovascular Invasion	9 (43%)	0.63±0.25	0.012
Differentiation			
Well	4 (17%)	0.00—Referent	—
Moderate/Poor	10 (44%)	1.09±0.34	0.001
Unknown	9 (39%)	1.07±0.29	<0.001
*Anthropometrics*			
BMI, kg/m^2^	30.2±5.5	0.07±0.02^b^	0.001
Visceral Adipose Tissue, cm^2^	127.4±56.8	0.08±0.04^c^	0.036
*Plasma Concentrations*			
Insulin	2.70±0.55	0.06±0.02^d^	0.011
IGF-1	4.03±0.23	-0.01±0.05^d^	0.763
IGFBP-3	7.62±0.26	-0.09±0.05^d^	0.054
sICAM-1	5.82±0.60	0.04±0.02^d^	0.037
sVCAM-1	6.91±0.38	0.06±0.03^d^	0.045

^a^Per 5 years of age

^b^Per 1 kg/m^2^ of BMI

^c^Per 20 cm^2^ of visceral adipose tissue

^d^Per 0.1 unit increase in log-transformed geometric mean

Baseline characteristics of the 23 participants were balanced across randomized groups, with exception of insulin (*P* = 0.053) and sICAM-1 (*P* = 0.049; **Table 2**). Average CTCs at baseline were not statistically significantly different among the three groups (*P* = 0.404), though the control group had numerically fewer CTCs compared with the two exercise groups (**Table 3**). Categorically, the proportion of patients with ≥1 (*P* = 0.657), ≥2 (*P* = 0.236), and ≥3 (*P* = 0.740) CTCs were similar among the three groups.

**Table 2:** Characteristics between study groups at baseline.

**Characteristic**	**Control (*n* = 5)**	**Low-Dose (*n* = 11)**	**High-Dose (*n* = 7)**	***P***
*Demographic*				
Age, years	55.6±4.3	58.4±9.6	52.1±11.2	0.399
Sex, %				
Male	0 (0%)	5 (45%)	2 (29%)	0.220
Female	5 (100%)	6 (55%)	5 (71%)	
Race, %				
White	4 (80%)	9 (82%)	6 (86%)	0.999
Black/Other	1 (20%)	2 (18%)	1 (14%)	
Smoking History, %				
Never	3 (60%)	4 (36%)	4 (57%)	0.641
Former/Current	2 (40%)	7 (64%)	3 (43%)	
*Tumor & Treatment*				
Stage, %				
I	0 (0%)	2 (18%)	1 (14%)	0.826
II	1 (20%)	4 (36%)	3 (43%)	
III	4 (80%)	5 (45%)	3 (43%)	
T Stage, %				
T1-T2	0 (0%)	2 (18%)	1 (14%)	0.924
T3	3 (60%)	7 (64%)	4 (57%)	
T4	2 (40%)	2 (18%)	2 (29%)	
N Stage, %				
N0	1 (20%)	6 (55%)	4 (57%)	0.484
N1-2	4 (80%)	5 (45%)	3 (43%)	
Chemotherapy, %	5 (100%)	7 (64%)	5 (71%)	0.390
Time Since Treatment Completion, Months	8 [3–12]	7 [4–11]	9 [5–17]	0.761
Lymphovascular Invasion	1 (20%)	5 (56%)	3 (43%)	0.470
Differentiation				
Well	1 (20%)	3 (27%)	0 (0%)	0.659
Moderate/Poor	2 (40%)	5 (45%)	3 (43%)	
Unknown	2 (40%)	3 (27%)	4 (57%)	
*Anthropometrics*				
BMI	29.3±4.9	28.6±3.9	33.3±7.3	0.366
Visceral Adipose Tissue	97.4±53.4	123.4±43.1	154.9±71.8	0.299
*Plasma Concentrations*				
Insulin, pmol/L^a^	2.33±0.24	2.75±0.37	2.88±0.82	0.053
IGF-1, ng/mL^a^	4.01±0.28	4.03±0.20	4.04±0.27	0.987
IGFBP-3, ng/mL^a^	7.49±0.16	7.59±0.21	7.74±0.36	0.304
sICAM-1, ng/mL^a^	5.14±0.64	6.05±0.31	5.95±0.64	0.049
sVCAM-1, ng/mL^a^	6.71±0.33	7.08±0.41	6.77±0.23	0.152

^a^Log-transformed geometric mean

**Table 3:** Effects of exercise on CTCs.

**CTC count per 1 mL blood**	**Control**	**Low-Dose**	**High-Dose**
Baseline, Mean ± SD	1.8±3.5	3.8±3.5	3.7±2.8
Change, LS Mean ± SE	−0.59±0.56	−1.34±0.34	−1.18±0.40
*P*	0.292	<0.001	0.004

Compared with baseline, statistically significant decreases in CTCs were observed in the low-dose (−1.34±0.34; *P*<0.001) and high-dose (−1.18±0.40; *P* = 0.004) exercise groups, whereas no significant change was observed in the control group (−0.59±0.56; *P* = 0.292). When we adjusted for insulin or sICAM-1 (due to baseline imbalances), our effect estimates were not altered.

In longitudinal analyses that consolidated the three randomized groups, reductions in BMI (−0.07±0.02; *P* = 0.007), insulin (−0.08±0.03; *P* = 0.014), and sICAM-1 (−0.07±0.03; *P* = 0.005) were associated with reductions in CTCs (**Table 4**).

**Table 4:** Relationship between change in anthropometric and plasma concentrations and change in CTCs during six months among all participants (*n* = 23).

**Characteristic**	**Unit of Change**	**Δ in CTCs** **(LS Mean ± SE)**	***P***
BMI	−1.0 kg/m^2^	−0.07±0.02	0.007
Visceral Adipose Tissue	−20.0 cm^2^	−0.10±0.06	0.121
Insulin	−0.1^a^	−0.08±0.03	0.014
IGF-1	−0.1^a^	0.04±0.05	0.419
IGFBP-3	−0.1^a^	0.06±0.06	0.278
sICAM-1	−0.1^a^	−0.07±0.03	0.005
sVCAM-1	−0.1^a^	0.03±0.04	0.294

LS Mean, least squares mean; SE, standard error.

^a^Change per 0.1 unit decrease in geometric mean

## Discussion

In this pilot study, six months of moderate-intensity aerobic exercise at doses of 150 and 300 min·wk^-1^ among stage I-III colon cancer patients resulted in significant reductions from baseline in CTCs. In addition, reductions in BMI, insulin, and sICAM-1 were correlated with reductions in CTCs. Using the GEDI microfluidic platform, 78% of patients had ≥1 CTC, which is higher than previously published rates of 10–25% in stage I-III in colon cancer patients [3, 5, 6]. GEDI detects up to 400-fold more CTCs than alternative platforms [25], which has been utilized in prior studies in colon cancer and may have a greater dynamic range for detection and discrimination [3]. The results from this study may help to provide insight to potential new mechanisms through which exercise may improve disease outcomes among patients with colon cancer. The discussion herein outlines hypotheses about how exercise may reduce CTCs.

Exercise may have a direct effect on CTCs. We observed significant decreases in CTCs in the two exercise groups, whereas no significant decrease was observed among the control group. Experimental models of shear stress may provide insight to these findings. A shear stress of 0.4 dyn/cm^2^ reduces the viability and increases the amount of apoptotic cells in a colon cancer cell line when compared to static conditions [28]. Higher magnitudes of shear stress, 1–2 dyn/cm^2^, induces a more pronounced decrease in cell viability and increases apoptosis compared to static conditions, suggesting that the sensitization of cancer cells to apoptosis occurs in a dose-dependent fashion that is proportional to the magnitude of shear stress [28]. Moderate-intensity exercise in humans (at ~55% of the age-predicted maximum heart rate) produces shear stresses of 5.2–6.2 dyn/cm^2^ [13], and increases proportionally with exercise intensity [12]. In a colon cancer cell line, increasing the duration of exposure, from 10 to 120 minutes, at a fixed 2 dyn/cm^2^ shear stress, also reduces cell viability and increases apoptosis in a dose-dependent fashion that is proportional to the duration of shear stress exposure [28]. However, we did not observe a dose-response relationship with respect to volume, as 150 min·wk^-1^ reduced CTCs to a similar extent at 300 min·wk^-1^ of exercise. It is plausible that 150 min·wk^-1^ may be a sufficient exercise volume to reduce CTCs. These data suggest that exercise may reduce CTCs via mechanical forces. Our finding that exercise lowers CTCs is consistent with prospective cohort studies that suggest moderate or vigorous intensity exercise (high shear stress exposure) and larger volumes of exercise (long exposure to a given shear stress, e.g., 150 min·wk^-1^) are associated with a lower risk of disease recurrence [8]. These data may explain, in part, a direct biological or mechanical mechanism through which exercise reduces disease recurrence in colon cancer patients.

Exercise may have indirect effects on CTCs through alterations in the host or patient tumor microenvironment. We observed that reductions in BMI, insulin, and sICAM-1 were significantly correlated with reductions in CTCs over six months. *In vivo* and preclinical studies support these findings. Exposure of colon cancer cells to adipocytes and pre-adipocytes significantly increases cell proliferation [29], and in a mouse model, the surgical removal of visceral obesity reduces the development and slows the progression of colonic tumors and prolongs survival [30]. Hyperinsulinemia increases colon cancer cell resistance to 5-fluorouracil [31], and oxaliplatin chemotherapy [31, 32], and exposure to insulin promotes colonic tumor multiplicity [33]. sICAM-1 is associated with obesity and hyperinsulinemia [34], and exposure to sICAM-1 stimulates tumor growth [35], and the inhibition of sICAM-1 attenuates colonic tumor cell invasion [36]. These *in vivo* and preclinical data are supported by prospective cohort studies that suggest higher BMI and visceral obesity [16, 18], hyperinsulinemia [17], and elevated sICAM-1 [37] are associated with recurrence and survival among patients with colon cancer. Exercise reduces visceral obesity in linear dose-response fashion [19], and insulin and sICAM-1 are reduced in nonlinear fashion [20, 23]. Collectively, these data may explain, in part, an indirect biological mechanism or mediator of exercise, such that the host tumor microenvironment is favorably altered by reducing available growth factors and improvement of immune system function [15], suppressing the growth of tumors that give rise to CTCs, and potentially reducing disease recurrence in colon cancer patients.

Additional research is needed to replicate our hypothesis-generating findings and begin to provide clinical context to these data. Changes in CTCs in response to an intervention, such as exercise, might represent an intermediate efficacy endpoint [38]. In the metastatic setting, changes in CTCs often precede radiologic progression, increasing the promise of CTCs for use as an endpoint [38]. However, less is known about the use of CTCs in the early-stage setting, and fundamental questions remain unanswered. It is unknown how many CTCs are needed to classify a patient at high-risk for disease recurrence. Studies have used thresholds of ≥1, ≥2, ≥3 CTCs to classify patients at high-risk of disease recurrence. While CTCs in the early-stage setting have been shown to be prognostic, it is unknown if reducing CTCs lowers the risk of disease recurrence (i.e., are CTCs a valid surrogate of disease recurrence and overall survival).

If CTCs are valid as a surrogate endpoint in the early-stage setting [39, 40], then studies are needed to determine which lifestyle behaviors best reduce CTCs, and the minimal required reduction in CTCs that is necessary to lower the risk of disease recurrence. Examining the comparative importance of reducing CTCs through mechanical forces (i.e., exercise) versus altering the tumor microenvironment exclusively (i.e., weight loss through caloric restriction) is a reasonable line of inquiry. It is plausible that the combination of exercise and weight loss may possess synergistic effects to reduce CTCs through the combination of mechanical forces from exercise and alterations in the tumor microenvironment from weight loss. Studies are needed to examine this hypothesis. The pairing of lifestyle interventions with pharmacological therapies such as aspirin, which inhibits platelet aggregation decreasing the ability for CTCs to bind to the endothelium (i.e., altering the mechanical efficiency of extravasation) [41], or metformin to reduce insulin (i.e., altering the host tumor microenvironment through growth factor suppression) [42], warrant investigation as synergy may exist between two complementary interventions.

There are several limitations to this study. The size of our study population was very small, which reduces the reliability of our findings. Because of the limited availability of GEDI chips, only 23 of 39 participants were analyzed, which may compromise randomization. We acknowledge that the control group had numerically fewer CTCs at baseline. Studies with larger sample sizes are needed to confirm that exercise reduces CTCs. This study should be interpreted with hypothesis-generating implications. We did not recruit patients based on having enumerable CTCs at baseline. Study participants were younger than the population from which they were recruited [21]. Therefore, the finding that older age at baseline was associated with fewer CTCs may be a statistical artifact of our study cohort. Alternatively, because of the enrichment with younger individuals in our sample, our study may overestimate the prevalence of CTCs in the population (78%). We tested a moderate intensity of exercise (50–70% of the age predicted maximum heart rate) at two distinct volumes (150 and 300 min∙wk^-1^). It is unknown how varying the exercise intensity would alter CTCs. We studied the chronic effects of exercise. It is unknown if reductions in CTCs occur after an acute bout of exercise or only after chronic exercise training. It is unknown what happens to CTCs upon the cessation of exercise. We are unable to determine the source of CTCs. It is unknown if the identified CTCs were shed from the primary tumor prior to surgical resection or are from established distant micro-metastatic foci that are not yet clinically detectable. The median time from finishing cancer therapy in our sample was eight months, therefore both scenarios are plausible. As this was considered an exploratory and hypothesis-generating study, we did not adjust our type I error rate for multiplicity.

There are several strengths to this study. We used the GEDI microfluidic platform, which has a high sensitivity to detect CTCs [25]. The enumeration of CTCs was completed by a technician who was blinded to treatment assignment. Adherence to both exercise doses of exercise was 90% and confirmed with objective monitoring. Most our study sample received chemotherapy, suggesting the population was at elevated risk of experiencing disease recurrence. Therefore, the population sample is ideal to study the effects of exercise on CTCs.

In conclusion, exercise may lower CTCs in patients with resected stage I-III colon cancer. The findings from this pilot study may be useful to begin to unravel the biologic or biobehavioral mechanisms through which exercise may reduce the risk of disease recurrence among patients with colon cancer. This hypothesis-generating observation warrants further investigation and replication.

## Supporting information

S1 Checklist CONSORT 2010 checklist. (DOC)

S1 Table Comparison between full study sample and CTC subsample at baseline. (DOCX)

S1 Protocol Study protocol. (DOCX)

S1 Data Public data (limited dataset). (XLS)
