# Supplementary material for: Effects of exercise on circulating tumor cells among patients with resected stage I-III colon cancer
Source: PLoS One. 2018 Oct 17;13(10):e0204875. doi: 10.1371/journal.pone.0204875 (PMC6192582; doi:10.1371/journal.pone.0204875)
Supplement: S1 Table — (DOCX) [file pone.0204875.s002.docx]

**S1 Table**. Comparison between full study sample and CTC subsample at baseline

| Characteristic | Full Study  Sample (*N*=39) | CTC Subsample  (*n*=23) | *P* |
| --- | --- | --- | --- |
| *Demographic* |  |  |  |
| Age, years | 56.5±9.9 | 55.9±9.3 | 0.819 |
| Sex, % |  |  |  |
| Male | 15 (38%) | 7 (30%) | 0.318 |
| Female | 24 (62%) | 16 (70%) |  |
| Race, % |  |  |  |
| White | 31 (79%) | 19 (83%) | 0.694 |
| Black/Other | 8 (21%) | 4 (17%) |  |
| Smoking History, % |  |  |  |
| Never | 23 (59%) | 11 (48%) | 0.111 |
| Former/Current | 16 (41%) | 12 (52%) |  |
| *Tumor & Treatment* |  |  |  |
| Stage, % |  |  |  |
| I | 5 (13%) | 3 (13%) | 0.985 |
| II | 14 (36%) | 8 (35%) |  |
| III | 20 (51%) | 12 (52%) |  |
| T Stage |  |  |  |
| T1-T2 | 6 (15%) | 3 (13%) | 0.565 |
| T3 | 25 (64%) | 14 (61%) |  |
| T4 | 8 (21%) | 6 (26%) |  |
| N Stage |  |  |  |
| N0 | 19 (49%) | 11 (48%) | 0.999 |
| N1-2 | 20 (51%) | 12 (52%) |  |
| Chemotherapy, % | 28 (72%) | 17 (74%) | 0.734 |
| Time Since Treatment Completion, Months | 10 [5−16] | 8 [4−15] | 0.059 |
| Lymphovascular Invasion | 13 (36%) | 9 (43%) | 0.484 |
| Differentiation |  |  |  |
| Well | 7 (18%) | 4 (18%) | 0.878 |
| Moderate/Poor | 18 (46%) | 10 (43%) |  |
| Unknown | 14 (36%) | 9 (39%) |  |
| *Anthropometrics* |  |  |  |
| BMI, kg/m^2^ | 30.3±5.8 | 30.2±5.5 | 0.954 |
| Visceral Fat Area, cm^2^ | 132.1±54.9 | 127.4±56.8 | 0.568 |
| *Plasma Concentrations* |  |  |  |
| Insulin, pmol/L^a^ | 2.78±0.58 | 2.67±0.55 | 0.198 |
| IGF-1, ng/mL^a^ | 4.07±0.27 | 4.03±0.23 | 0.032 |
| IGFBP-3, ng/mL^a^ | 7.55±0.32 | 7.62±0.26 | 0.189 |
| sICAM-1, ng/mL^a^ | 5.81±0.56 | 5.82±0.60 | 0.864 |
| sVCAM-1, ng/mL^a^ | 6.88±0.32 | 6.91±0.38 | 0.786 |

^a^Log-transformed geometric mean. *P* values represent the test of differences between the 23 participants with a CTC sample and the 16 participants without a CTC sample.
