# Supplementary material for: Effects of exercise on circulating tumor cells among patients with resected stage I-III colon cancer
Source: PLoS One. 2018 Oct 17;13(10):e0204875. doi: 10.1371/journal.pone.0204875 (PMC6192582; doi:10.1371/journal.pone.0204875)
Supplement: S1 Protocol — (DOCX) [file pone.0204875.s003.docx]

**Application for Review of Human Research: IRB Protocol Summary**

**Biomedical Research Section II**

***Principal Investigator*:** Kathryn H. Schmitz, PhD, MPH

**PROTOCOL TITLE**

***1. Full Title***

The COURAGE trial: Colon Recurrence and Aerobic Exercise: A Feasibility Study.

***2. Brief Title***

Exercise and Colon Cancer.

**STUDY SPONSORSHIP**

***1. Funding Sponsor***

National Institutes of Health (NIH), National Cancer Institute (NCI): R21 CA182767

National Institutes of Health (NIH), National Cancer Institute (NCI): F31 CA192560

***2. Primary Sponsor***

***Principal Investigator*:** Kathryn H. Schmitz, PhD, MPH, University of Pennsylvania (R21 CA182767)

Justin C. Brown, MA, University of Pennsylvania (F31 CA192560)

***Program Officer:*** Frank Perna, PhD, National Cancer Institute (R21 CA182767)

Jeanette Korczak, PhD, National Cancer Institute (F31 CA192560)

**PROTOCOL ABSTRACT**

Despite the success of surgery and chemotherapy among people with colon cancer, 30-50% of patients develop recurrent disease. Physical activity has emerged as a potential lifestyle intervention to reduce cancer recurrence and improve survival among people with colon cancer (CC). This pilot study aims to identify the dose-response effects of aerobic exercise on molecular and cellular pathways associated with physical activity and CC outcomes among patients with stage II and III CC.

**OBJECTIVES**

***1. Overall Objectives***

We will compare the feasibility and safety of two doses of aerobic exercise among 39 CC survivors, while simultaneously exploring the potential biological effects. We aim to compare 150 min∙wk^-1^ and 300 min∙wk^-1^ of moderate-intensity aerobic exercise to a wait-list control group. The findings from this study will help to establish the feasibility and safety of the dose-response effects of exercise among CC survivors and provide effect size estimates to inform the development of a phase III trial.

**Primary aim:**

To conduct a dose-response randomized exercise intervention consisting of 150 or 300 min∙wk^-1^ of moderate intensity aerobic exercise or wait-list control among people with CC. We aim to quantify and describe:

1. the feasibility of prescribing 150 or 300 min∙wk^-1^ of aerobic exercise;
2. the incidence of symptoms or events requiring alteration of aerobic exercise dose;
3. the biological efficacy of aerobic exercise on soluble intercellular adhesion molecule-1 (sICAM-1), and soluble vascular adhesion molecule-1 (sVCAM-1) prognostic biomarkers;

Hypothesis: exercise will favorably reduce sICAM-1 and sVCAM-1 in dose-response fashion.

**Secondary aims:**

To quantity and describe the biological efficacy of aerobic exercise on visceral adipose tissue, measured with a novel and validated method using dual energy x-ray absorptiometry

Hypothesis: exercise will favorable reduce visceral adipose tissue in dose-response fashion.

To quantity and describe the dose-response effects of moderate-intensity aerobic exercise on levels of fasting insulin.

Hypothesis: We hypothesize exercise will favorably reduce levels of fasting insulin in dose-response fashion.

**Exploratory aims:**

To enumerate circulating tumor cells (CTCs) before and after exercise, and describe their relationship with changes in sICAM-1 and sVCAM-1.

Hypothesis: We hypothesize 50% of study participants will have detectable CTCs, and that aerobic exercise training will result in a decrease in the number of CTCs in a dose-response fashion, and this reduction will be correlated with sICAM-1 and sVCAM-1 levels.

To quantity and describe the relationship between the exercise-induced improvements in visceral adipose tissue and the exercise-induced improvements fasting insulin.

Hypothesis: We hypothesize the exercise-induced improvements in visceral adipose tissue will be positively and linearly correlated with the exercise-induced improvements in fasting insulin.

***2. Primary Outcome Variable(s)***

The primary outcome variables that will be used to support the study objective as related to the biological efficacy of exercise will include:

sICAM-1 and sVCAM-1 measured at baseline and six-months.

***3. Secondary Outcome Variable(s)***

Secondary outcome variables as they relate to our primary aim include:

1. exercise adherence, quantified as the percentage of total dose completed relative to the total dose prescribed during the six-month study, and;
2. adverse events (symptoms or events), requiring alteration (or cessation) of the prescribed aerobic exercise dose during the six-month study.
3. visceral adipose tissue measures at baseline and six-months.
4. fasting insulin measured at baseline and six-months.

Exploratory Outcome Variable(s):

1. CTCs measured at baseline and six-months.

**BACKGROUND**

**Colon Cancer Treatment and Prognosis.** There are 103,000 people diagnosed annually with colon cancer (CC) in the United States.[1] Among those diagnosed, 39% will have localized CC (confined to the primary site), 36% will have regional CC (spread to regional lymph nodes), and 20% will have metastatic disease (spread to distant organs).[1] Among those without metastatic disease, 5-year survival rates for localized and regional CC are 90% and 70%, respectively.[1] Surgery is the primary treatment modality for local and regional CC, with curative resection occurring in 80-85% of patients.[2] Those with regional disease may also receive adjuvant chemotherapy.[3,4] Despite the efficacy of resection and adjuvant chemotherapy, 30-50% of patients with local and regional CC develop recurrent cancer.[2,5] Eighty percent of recurrences occur in the first three years after treatment, and 91% of patients who develop a recurrence by three years, die before five years.[6] The high rate of recurrent disease is a critical barrier to promote the health and longevity of CC survivors. An approach to reduce this barrier is to develop interventions to minimize recurrence that CC survivors can accomplish autonomously, such as lifestyle and behavioral modification.

**Colon Cancer Recurrence.** Cancer recurrence and metastasis are the leading causes of morbidity and mortality in CC, marking the transition from localized, potentially curable to disseminated, usually incurable disease.[7,8] CC recurrence is typically characterized by locoregional relapse and/or distant metastasis to the liver or lung.[5] A key condition for CC recurrence is that existing micro-metastases or circulating tumor cells (CTCs) be present in the body at the time of curative treatment.[8,9] Existing micro-metastases have been difficult to characterize given their absence with radiographic imaging and with other forms of staging. Autopsy studies suggest 66% of metastases in CC are explained by vascular blood flow alone.[9] Given the challenge to identify micro-metastases, characterizing biomarkers that promote existing tumor growth and dissemination of CTCs in the vasculature may advance knowledge of CC recurrence and progression. For example, 25-55% of non-metastatic CC survivors have measureable volumes of CTCs in their blood after completing curative treatment.[10] The presence of CTCs predicts recurrence, tumor progression, and survival among CC survivors.[11-16] Among 438 patients with local or regional CC (stages I-III), 31% had tumor cells in peripheral blood after completing treatment, and the presence of CTCs was associated with a 29-fold increase in recurrence (p<0.001).[12] The implementation of CTC technology into clinical care has been subject to debate.[17] Little evidence currently exists to leverage knowledge about CTCs to improve clinical outcomes.[17] However, many of these previously published reports documenting relatively low concentration of CTCs in patients with early colon cancer and in those with frank metastases utilized the CellSearch^®^ system to quantify CTCs, the first FDA-approved device to identify CTCs.[18,19] We have developed an alternative detection technology, GEDI (geometrically enhanced differential immunocapture), which has been shown to be superior to CellSearch^®^ in head-to-head studies.[20] In CC, we have been able to detect up to 100-fold greater CTCs compared to CellSearch^®^ (unpublished data), and approximately 80% of patients with advanced CC contained CTCs. Thus, our innovative approach provides unsurpassed sensitivity in quantifying CTCs. Underscoring this sensitivity, we have detected circulating epithelial cells (CECs) in the bloodstream of patients at high-risk for tumor formation, yet do not have a diagnosis of cancer. In mouse models of spontaneous cancer, contrary to current models, CECs and CTCs can seed the bloodstream prior to the formation of clinically (radiographic) detectable tumors.[21] Seeding of the blood circulation by CECs also occurs in patients in the absence of clinically (radiographic) detectable tumors. We and others have shown that CECs can be detected in the bloodstream of patients with advanced precancerous adenomas of the colon[22] (and unpublished). This is of interest since these are patients with no diagnosis of cancer, but may harbor occult neoplastic disease. Based on our results, it is likely that elaboration of CTCs occurs early in the recurrent metastatic cascade. The sources of CTCs are likely distant micro-metastatic foci. We hypothesize that cancer cells disseminate via the circulation during the earliest stages of recurrent metastatic growth, mirroring what occurs during the primary tumor setting. If this does indeed occur, we are uniquely situated to detect this phenomenon using the GEDI device. By implementing the GEDI technology in our study, we will have preliminary data to suggest if exercise is one such intervention to improve outcomes by acting through cell-adhesion and other pathways implicated at the intersection of CC recurrence and exercise.[23-26] If our hypothesis is correct that exercise will serve to delay or inhibit the progression of recurrent or metastatic CC, this may be reflected in CTC concentration. In conclusion, identifying and intervening on pathways that act to promote the growth of existing micro-metastases and stimulate CTC activity may be one means to reduce recurrent disease among CC survivors.[7-9]

**Colon Cancer and the sICAM-1 and sVCAM-1 Pathway.** Cell-adhesion molecules promote the growth and progression of existing micro-metastases,[27,28] and promote CTC differentiation, contact inhibition, and apoptosis.[29,30] The immunoglobulin superfamily is a specific group of cell-adhesion molecules that has been identified to play a role in the development and recurrence of CC.[7,30,31] Two specific proteins in the immunoglobulin superfamily, soluble intercellular adhesion molecule-1 (sICAM-1), and soluble vascular adhesion molecule-1 (sVCAM-1) have been found in the blood vessels of CC tumors and identified as pivotal in the recurrence and progression of CC tumors.[32] sICAM-1 and sVCAM-1 expression is stimulated by proinflammatory cytokines. These cytokines trigger the PI3K-Akt pathway, resulting in the activation of mitogen-activated protein kinase (MAPK), nuclear factor kappa-b (NF-κB), c-Jun N-terminal kinases (JNK), P38, and P44/42 enzyme expression, with the end product of this cascade being the expression of sICAM-1 or sVCAM-1.[7,33,34] Numerous points along this pathway have been associated with CC recurrence. For example, PI3K-Akt expression is known to increase tumor invasiveness,[35] migration, and progression.[36] NF-κB is known to be a mediator of tumor surveillance, and progression.[37]

sICAM-1 is not expressed in healthy colonic cells, and over expressed in CC tumor cells.[31,38] sICAM-1 expression increases in dose-response fashion with tumor growth and progression,[27] and predicts survival, lymph node and distant metastases, and is associated with serum carcinoembryonic antigen (CEA), the clinical biomarker used to monitor CC recurrence and tumor progression.[39] Among stage II CC survivors, elevated sICAM-1 levels were associated with an 8.8-fold increase in risk of mortality (p=0.001).[39] The relationship between sICAM-1 and CC is well-established as other investigative groups have reached similar conclusions about the predictive significance of sICAM-1 and CC.[27,32,40] Similar to sICAM-1, sVCAM-1 is expressed in dose-response fashion and predicts tumor growth and progression.[27,41] sVCAM-1 is chronically elevated for at least one-month after surgical resection for CC, which may promote growth of micro-metastases and aberrant CTC behavior immediately after surgery.[42] An animal model confirmed the intraperitoneal microenvironment after surgery enhances the activity of disseminated CTCs as hypothesized.[43] sVCAM-1 additionally predicts survival,[27] recurrence,[44] lymph node and distant metastases.[27,45] Among 100 stage I-IV CC survivors, elevated sVCAM-1 levels were associated with an 11.1-fold increase in liver metastasis (p=0.028).[45] The importance of sVCAM-1 and CC as described above has been observed by others.[27,32,40-42,44,46]

Prior literature supports the hypothesis that sICAM-1 and sVCAM-1 are active molecules of the cancer recurrence cascade, specifically associated with growth and progression of existing micro-metastases in melanoma,[47] CC,[27] and in in vivo studies.[48] sICAM-1 and sVCAM-1 play important roles in promoting CTC extravasation to distant tissues and recruitment of pro-tumorigenic leukocytes to existing micro-metastases. sICAM-1 and sVCAM-1 cleavage and release into the circulation occurs during upregulation and engagement of inflammatory and proangiogenic factors in cells with receptors specific to these molecules, i.e., at the earliest stages of dissemination at the distant organ sites.[49] We hypothesize that exercise may inhibit both seeding of distant organs by CTCs and the cultivation of the angiogenic milieu that is thought to be required for metastatic tumor growth. As a first step towards addressing this hypothesis, we will measure serum sICAM-1 and sVCAM-1 given their importance in mediating these processes.

**Colon Cancer and Visceral Adipose Tissue.** The two main compartments in which adipose tissue is stored include: 1) subcutaneous adipose tissue (SAT) and 2) VAT.[50,51] SAT represents a usual physiologic buffer to store excess energy.[51] When the storage capacity of SAT is exceeded, VAT is accrued. Accordingly, 80–90% of all body fat is SAT, and the remaining 10–20% is VAT.[52] The cells that constitute adipose tissue are known as adipocytes. The adipocytes that constitute VAT are physiologically and pathologically distinct from SAT.[51,52] The adipocytes of VAT are more deleterious to health than the adipocytes of SAT, due to their metabolic secretion of bioactive compounds such as adipokines, cytokines, hormone-like factors, and other metabolites.[53,54] The deleterious metabolic activity of VAT, coupled with anatomic proximity to vital organs, and direct drainage into portal venous vasculature (a location of many CC recurrences), manifests as a pro-tumorigenic milieu.[55]

VAT independently predicts poor CC outcomes.[56] Six observational epidemiologic studies have examined VAT and CC outcomes; four studies used computed tomography (CT) imaging,[57-60] and two studies used waist circumference as a proxy measure of VAT.[61,62] All six studies concluded that higher levels of VAT predict poor CC outcomes such as disease-free survival, cancer-specific mortality, and all-cause mortality. For example, among 74 stage II CC survivors, above-median values of VAT predict a 3.76-fold increase in recurrence (*P*=0.015), and 2.72-fold increase in recurrence and/or death from any cause (i.e., disease-free survival; *P*=0.032).[57] Among 526 CC survivors, each 10-centimenter increase in waist circumference predicts a 20% increase in recurrence and/or death from any cause (*P*=0.008).[61] VAT is a strong independent predictor of all-cause mortality among older adults without a history of cancer. Among 1,089 men and women aged 18–84, each standard deviation increase in VAT (i.e., 70.1 cm^2^), predicts a 74% increase in all-cause mortality, independent of age, smoking status, and cardiovascular disease (*P*<0.001).[63] The relationship between VAT and mortality is j-shaped, such that as VAT increases, mortality increases exponentially.[64]

Body mass index (BMI), derived by dividing weight (in kilograms) into height (in meters) squared (kg/m^2^), is a widely used metric to quantify body composition. BMI is a predictor of incident CC,[65] however several studies have concluded that BMI does not influence CC outcomes after diagnosis.[66] For example, among 1,053 stage III CC survivors, BMI at diagnosis did not predict CC recurrence (*P*_trend_=0.86), disease-free survival (*P*_trend_=0.65), or all-cause mortality (*P*_trend_=0.63).[67] These findings are in clear contrast to the consistent deleterious impact of VAT on CC outcomes, as described above. It is known that BMI and VAT are poorly correlated.[60] For example, a patient may have a “healthy” BMI, but have pathogenic levels of VAT, or vice-versa. Calculation of BMI relies on the assumption that adipose tissue is distributed uniformly across the body, ignoring the known heterogeneity of regional deposition of adiposity.[52] There is substantial inter-individual variation between BMI and VAT; at a given BMI unit (i.e., 30.0 kg/m^2^), VAT may vary by 40%.[68] Among CC survivors, BMI accounts for only 30.4% of the variability in VAT.[57] Collectively, these data indicate that the location of adiposity (i.e., in the viscera) is more influential on CC outcomes than the quantity of total-body adiposity (i.e., BMI).[52,61] This observation is consistent with the hypothesis that VAT is a metabolically active tissue that lends rise to deleterious CC outcomes.[51] VAT associates with hyperinsulinemia among CC survivors (*r*=0.519; *P*<0.001),[69] and is implicated in the progression of recurrent and metastatic CC.[23,70]

**Colon Cancer and Hyperinsulinemia.** Hyperinsulinemia is characterized by a high concentration of insulin and exaggerated insulin response to increases in glucose concentration.[70] The adipocytes of VAT are more insulin-resistant than the adipocytes of SAT.[51] VAT is positively associated with elevated levels of fasting insulin,[71] and accounts for 18–40% of the variability in levels of fasting insulin (*P*<0.001).[72] Each 10-centimer increase in VAT area (cm^2^) predicts a 0.46 *μ*U/mL increase in fasting insulin.[73] VAT is an independent predictor of insulin sensitivity (i.e., how efficient the body uses insulin to lower blood glucose).[74] The relationship between VAT and insulin is meaningful for CC survivorship because CC cells have insulin receptors and proliferate when exposed to insulin.[23] Insulin circulates systemically in the vasculature, and is therefore capable of promoting the progression of local recurrence and distant metastases among CC survivors. CC survivors have fasting insulin levels that are 58% higher than age- and gender-matched controls without a history of CC (*P*<0.001).[69] Hyperinsulinemia is often a symptom among people diagnosed with early stage type 2 diabetes mellitus (T2DM). CC survivors with T2DM have significantly worse prognosis than those without T2DM.[75-78] For example, among 3,759 stage II/III CC survivors, those with T2DM had shorter 5-year disease-free survival (48% vs. 59%; *P*<0.001), and shorter 5-year overall survival (57% vs 66%; *P*<0.001), respectively.[75] Markers of insulin secretion, such as C-peptide, predict poor CC outcomes. Among 373 stage I-III CC survivors, high levels of C-peptide predict a 1.87-fold increase in all-cause mortality.[79] Hyperinsulinemia also promotes insulin growth factor (IGF)-I biosynthesis, which inhibits production of insulin-like growth factor binding protein (IGFBP)-I, allowing insulin to remain chronically elevated.[70] Low levels of IGFBP-I predict a 2.3-fold increase in CC-specific and all-cause mortality among stage I-III CC survivors.[79,80] Collectively, this evidence supports the hypothesis that hyperinsulinemia is a predictor of recurrence and metastasis among CC survivors. Two ongoing studies examine the impact of exercise on insulin among CC survivors.[81,82] These studies are limited in that they test a single dose of exercise, and neither quantify VAT using objective imaging methods, electing to use waist circumference measures as a proxy for VAT. The symbiotic relationship between VAT and hyperinsulinemia yield a pro-tumorigenic environment which lends rise to recurrent and metastatic disease among CC survivors.[55] Interventions that collectively reduce VAT and insulin may therefore have considerable impact on the quality and longevity of CC survivorship.

**Colon Cancer and Physical Activity**. Increasing levels of post-diagnosis physical activity (PA) are associated with a 42% improvement in disease-free survival,[24] 40% reduction in cancer recurrence,[24] and 45-71% reduction in CC-specific mortality.[24-26] The evidence also suggests PA improves outcomes in dose-response fashion, such that CC survivors who engage in larger volumes of PA have more favorable disease free survival (p_trend_=0.01), recurrence (p_trend_=0.03), and CC-specific mortality (p_trend_=0.008).[24-26] The relationship between PA volume and CC outcomes has been depicted as a linear or quadratic pattern using smoothed splines.[24,26] The splines suggest 225 min∙wk^-1^ may be the minimum threshold necessary to improve CC outcomes, as is prescribed in current ongoing studies.[81,82] However CC outcomes continue to improve beyond 225 min∙wk^-1^, suggesting that larger doses may be more efficacious.[24,26] PA volume >400 min∙wk^-1^ does not appear to confer additional improvements in these outcomes. Categorically, a dose of ~300 min∙wk^-1^ of moderate-intensity PA appears necessary to reduce recurrence, improve disease-free survival, and improve CC-specific mortality.[24-26] It remains unknown if larger doses of exercise are behaviorally feasible and have tolerable safety and symptom profiles.[24] Interestingly, levels of PA before diagnosis of CC do not associate with CC-specific mortality.[25,61,83] This suggests the hypothesized pathways in which PA associate with primary prevention of CC may differ from that of secondary prevention of CC.[84] The American College of Sports Medicine (ACSM) and the American Cancer Society published guidelines for cancer survivors, recommending 150 min∙wk^-1^ of moderate intensity PA.[85,86] This recommendation is inconsistent with the dose of PA necessary to reduce recurrence, and improve CC-specific mortality indicated by the epidemiologic literature. Several reviews, including The ACSM statement for exercise among cancer survivors, have called for more research to empirically investigate the dose-response effects of exercise,[86-89] and the safety profile of prescribing larger doses of exercise.[86,90] Among 295 breast cancer survivors who participated in our PAL trial,[91,92] a yearlong weight-lifting study, 21% required a dose-modification or cessation of weight-lifting as a result of a health event.[93] These findings parallel evidence from adults aged ≥70 years in an exercise trial, where 34% of participants had a healthcare event that influenced their completion of the initial prescribed dose of exercise.[94] The insufficient evidence regarding the specific dose of exercise necessary to improve CC outcomes likely serves as a barrier to the dissemination of exercise to CC survivors. Clinicians have noted they do not know what tell their patients about exercise.[95] The majority of clinicians (57%) provide no recommendation about PA.[96,97] Obtaining systematic data pertaining to dose-reduction or cessation of exercise is critical to future optimization of the allocation of behaviorally intensive resources, such as cancer rehabilitation and exercise programming. These data may be fruitful as exercise moves towards the forefront for consideration as a standard of care for cancer survivors, similar to cardiac rehabilitation.[96]

**Exercise and sICAM-1 and sVCAM-1 Pathway.** Elevated sICAM-1 and sVCAM-1 are associated with a two-fold increase of developing a future fatal cardiovascular event.[98] Prior studies hypothesized exercise may be a means to reduce sICAM-1 and sVCAM-1. Moderate-intensity aerobic exercise intervention studies have consistently reduced serum sICAM-1 and sVCAM-1 by 6-14%, and 12-30%, respectively, in a variety of populations including those with heart failure,[99] metabolic syndrome,[100] type 2 diabetes,[101,102] and peripheral artery disease.[103] The exercise prescription among these studies has been homogeneous, with most interventions increasing to a maximum of 150 min∙wk^-1^ of moderate-intensity walking or cycling over 8-26 weeks. However, using accelerometry to monitor PA, an epidemiologic study identified a dose-response relationship may exist for sICAM-1 and sVCAM-1, such that as PA volume increases, sICAM-1 and sVCAM-1 expression favorably decrease.[104] The interactions among sICAM-1, sVCAM-1, existing micro-metastases, and CTCs are unique to CC survivors. As such, to generalize findings from non-cancer populations could misrepresent the role of sICAM-1 and sVCAM-1 in CC recurrence.

**Exercise and Visceral Adipose Tissue.** Exercise is an efficacious modality to reduce VAT. The “Studies of a Targeted Risk Reduction Intervention through Defined Exercise” (STRRIDE) trial,[105] established a dose-response effect of exercise on VAT.[106,107] The STRRIDE study was a 4-arm randomized controlled trial that compared three-doses of exercise to a control group for six-months among 330 men and women who were overweight or obese with mild to moderate lipid abnormalities.[105] The STRRIDE study demonstrated a significant dose-response reduction in VAT, quantified using CT (*P*_trend_<0.001).[107,108] The dose-response effects of exercise on VAT have replicated empirically,[109] and in meta-analyses of randomized exercise trial using weighted regression models (*r*=–0.75; *P=*0.01).[110,111]

**Exercise and Hyperinsulinemia.** Exercise significantly reduces fasting insulin levels in dose-response fashion.[112-114] The STRRIDE study demonstrated a significant reduction in fasting insulin (*P*<0.001), and significant improvement in insulin sensitivity (*P*<0.05).[114] These experimental findings have been corroborated in a *post-hoc* analysis of a two-group randomized exercise study among overweight or obese post-menopausal women.[112] The observational epidemiologic data also support a dose-response relationship between exercise volume with fasting insulin,[115,116] and insulin sensitivity.[116] Exercise-induced improvements in VAT correlate with improvements in insulin sensitivity (*r*=–0.19; *P*=0.018),[107] and insulin resistance (*r*=0.66; *P*<0.01).[117]

**CHARACTERISTICS OF THE STUDY POPULATION**

***1. Target Population***

The target population for this study is adult (≥18 year old) stage II and stage III CC survivors (as defined below in the key inclusion criteria section), who have completed curative treatment (i.e., surgical resection and chemotherapy) for their CC.

Given that colon cancer affects both men and women, we will recruit both men and women with a history of a histologically confirmed colon cancer. We will aim to recruit 50% women within the proposed study. We will endeavor to recruit 25% minority recruitment within the proposed study. We will focus our minority recruitment efforts on African Americans, as the largest minority group in the Philadelphia metropolitan area. That said no minorities will be excluded from participation. Our approaches include targeted recruitment efforts and considerable outreach to succeed in these efforts.

***2. Accrual***

Accrual will be 39 total participants over 15-months, with an accrual goal of 2-3 per month. Available data indicates that 800 new CC patients will be diagnosed at the Abramson Cancer Center between April 2012 and March 2014. During the 15-month recruitment window, another 500 patients will be diagnosed. Of these 1300 patients, 715 (55%) will have stage II-III CC. We will seek to recruit 5% of these patients. In addition, we will recruit potentially eligible study participants who are outside the University of Pennsylvania Health System using the Pennsylvania and New Jersey state cancer registries.

The statistical justification for the sample size of this study is described in the statistical analysis portion of the study procedures section.

***3. Key Inclusion Criteria***

We propose to begin with three-months of relatively strict eligibility criteria (**Phase 1**). At the end of three-months, if we have not accrued at least 6 patients, eligibility criteria will be expanded for a second three-month recruitment phase (**Phase 2**). If, at the end of 6 months, we have still not accrued an average of 2 patients per month, we will further expand eligibility criteria (**Phase 3**).

1. **Phase 1 (Most Strict)** Eligibility criteria include:
   1. histologically confirmed TNM stage II-III CC;
   2. completed surgical resection and adjuvant chemotherapy (if applicable) within 1-24 months before entering the study;
   3. ≤120 min∙wk^-1^ of self-reported moderate or vigorous intensity PA using the Paffenbarger PA questionnaire;
   4. age ≥18 years;
   5. written physician approval;
   6. no additional surgery planned within the 6-month intervention (including colostomy reversal);
   7. ability to walk unaided for 6-minutes;
   8. no contraindications to exercise using the PA readiness questionnaire (PAR-Q), unless physician approves participation with specific knowledge of this contraindication.
2. **Phase 2 (less restrictive)** Eligibility criteria will loosen:
   1. the baseline physical activity level to <150 min∙wk^-1^ and;
   2. expand the time since completing surgical resection and adjuvant chemotherapy to 36-months before entering the study.
3. **Phase 3 (least restrictive)** Eligibility criteria will add:
   1. histologically confirmed TNM stage I CC and;
   2. expand to any time since completing surgical resection and adjuvant chemotherapy.

**Colorectal Cancer Staging TNM Criteria, AJCC 7^th^ Edition, 2009**

**Primary Tumor (T)**

TX: Primary tumor cannot be assessed

T0: No evidence of primary tumor

Tis: Carcinoma *in situ*: intraepithelial or invasion of lamina propria*

T1: Tumor invades submucosa

T2: Tumor invades muscularis propria

T3: Tumor invades through muscularis propria into the pericolorectal tissues

T4a: Tumor penetrates to the surfact of the visceral peritoneum**

T4b: Tumor directly invades or is adherent to other organs or structures***

*Note: Tis includes cancer cells confined within the glandular basement membrant (intraepithelial) or muscosal lamina propria (intramucosal) with no extension through the muscularis mucosae into the submucosa.

**Note: Direct invasion in T4 includes invasion of other organs or segments of the colorectum by way of the serosa; for example, invasion of the sigmoid colon by a carcinoma of the cecum.

***Note: Tumor that is adherent to other organs or stuctures, grossly, is classified as cT4b. However if not tumor is present in the adhesion, microscopically, the classification should be pT1-4a depending on the anatomical depth of wall invasion. The V and L classifications should be used to identify the presence or absence of vascular or lymphatic invasion whereas the PN site-specific factor should be used for perineural invasion.

**Regional Lymph Nodes (N)**

NX: Regional lymph nodes cannot be assessed

N0: No regional lymph node metastasis

N1: Metastasis in 1-3 regional lymph nodes

N1a: Metastasis in 1 regional lymph node

N1b: Metastasis in 2-3 regional lymph nodes

N1c: Tumor deposit(s) in the subserosa, mesentery, or nonperitonealized pericolonic or perirectal tissues without regional nodal metastasis.

N2: Metastasis in 4 or more regional lymph nodes

N2a: Metastasis in 4-6 regional lymph nodes

N2b: Metastasis in 7 or more regional lymph nodes

**Distant Metastasis (M)**

M0: No distant metastasis

**Stage Groupings (TNM)**

Stage I : T1 N0 M0

T2 N0 M0

Stage IIA: T3 N0 M0

Stage IIB: T4a N0 M0

Stage IIC: T4b N0 M0

Stage IIIA: T1-T2 N1, N1c M0

T1 N2a M0

Stage IIIB: T3-T2a N1, N1c M0

T2-T3 N2a M0

T1-T2 N2b M0

Stage IIIC: T3-T4a N2b M0

T4b N1-N2 M0

***4. Key Exclusion Criteria***

Ineligibility criteria for all three recruitment phases include:

1. history of another primary invasive cancer (other than non-melanoma skin-cancer);
2. evidence of metastatic CC (i.e., TNM M1);
3. planning to receive any additional adjuvant chemotherapy;
4. pregnant or breast feeding;
5. unable to provide baseline blood sample;
6. cardiac conditions, including the following:
   1. myocardial infarction or coronary revascularization procedure within prior 3 months;
   2. uncontrolled hypertension (systolic ≥180 mmHg or diastolic ≥100 mmHg);
   3. high-risk or uncontrolled arrhythmias;
   4. clinically significant valvular disease;
   5. decompensated heart failure;
   6. known aortic aneurysm;
7. any other condition that may impede testing of the study hypothesis or make it unsafe to engage in the exercise program (determined by the investigative team).

***5. Vulnerable Populations***

Children, pregnant women, fetuses, neonates, or prisoners are not included in this research study.

***6. Populations vulnerable to undue influence or coercion***

It is not known for a fact that all people that will be approached will not be vulnerable. Using the electronic medical records and the registry data we will screen out those who immediately do not quality (such that those who are less than 18 years old). In addition, patients in the UPHS system will have their oncologist approve our contacting of them before we do so. However, we cannot make any factual statements regarding those who we approach or do not approach.

We strive to ensure that all study participants are informed that the study is completely voluntary and their participation (or choice to not participate) will have no impact on their grades, employability, standing within the University, compensation, or future healthcare provision. All study staff members that interact with potential study participants are trained in human subjects and HIPAA practices.

Participants who require a surrogate consent due to cognitive impairment will be excluded due to concerns about appropriately following an exercise program which is partially unsupervised (exclusion criteria #6). This is done to ensure the safety of the person and to preserve the ability to accurately test the study hypothesis.

***7. Subject Recruitment***

Potentially eligible study participants will be identified using the electronic medical records and cancer registry at the University of Pennsylvania. After potentially eligible patients are identified, we will send a list of those patients to the attending oncologist asking them for permission to contact their patients about the study. All potentially eligible patients will receive recruitment letters that will be signed by their treating oncologist and study principal investigator to inform them about the trial. Mailed letters will be followed-up through telephone contact to assess study interest, and assess study eligibility.

Potentially eligible study participants will also be identified using the Pennsylvania and New Jersey state cancer registries. For potentially eligible study participants identified through the Pennsylvania and New Jersey state cancer registries (that is those not within the University of Pennsylvania Health System), we will send them a recruitment letter that will be signed by the principal investigator of the study to inform them about the trial.

In addition to targeted mailings, brochures describing the study will be disseminated to medical clinics (such as medical oncology and colorectal surgery) for their placement in patient waiting areas.

**STUDY DESIGN**

***1. Phase***

N/A

***2. Design***

This is a randomized controlled, single-blind, trial. Blinding will be ensured by reminding the participants not to reveal group at the beginning of their measurement visits.

***3. Study Duration***

The projected overall duration of the study, including the:

- estimated length of time to enroll all subjects and complete the study: 24 months
- length of a subject’s participation time in study: 7 months
- projected date of completion of the proposed study: May 31, 2016

**DRUGS OR DEVICES**

Drugs or devices are not used in this research study.

**STUDY PROCEDURES**

1. ***Procedures***

After obtaining written consent and written clearance from the primary care physician or oncologist is provided, a baseline measurement appointment will be scheduled. Measurements will include those described in detail below. All measurements described below will occur prior to randomization and at six-months. For all measurement visits, participants will be provided free parking or bus/subway tokens.

**Blood assays as outcomes.** Participants will undergo a fasting blood draw at baseline and six-months later at the same time of day through Penn’s CTSA-funded shared resource clinics. A total of 50.0 mL will be drawn per participant at baseline and again at six-months. All aliquots will be stored until the end of the study for analysis. Assays will be completed through the Human Immunology Core ((HIC) a shared resource of the Perelman School of Medicine, under the direction of Dr. Jean Boyer)). CTCs will be completed in the laboratory of Dr. Andrew Rhim at the University of Michigan (Co-I).

- sICAM-1 will be analyzed using an R&D systems enzyme linked immunoabsorbent assay.[99,104] The inter-assay coefficient of variation and sensitivity are 5.4%, and 0.049-0.254 ng/mL, respectively.[118]
- sVCAM-1 will be analyzed using an R&D systems enzyme linked immunoabsorbent assay. The inter-assay coefficient of variation and sensitivity are 7.7%, and 0.17-1.26ng/mL, respectively.[119] Both assays will be repeated in duplicate for quality control.
- CTCs from participant venous blood will be isolated using GEDI. GEDI was developed by Dr. Brian Kirby at Cornell University who has worked with Dr. Rhim extensively using this technique in a variety of populations, including prostate cancer,[120,121] and preneoplastic pancreas cancer.[21] GEDI is a microfluidic platform that utilizes antibody-coated obstacles to capture rare cells within blood. This allows for anticoagulated whole blood to be applied to the “chip” the size of a microscope slide in the laboratory using standard syringe pumps. To capture CTCs, obstacles will be coated with an antibody specific to epithelial cell-adhesion molecule (EpCAM), an epithelial cell-specific marker. After washes, captured cells will be stained with the nuclear marker DAPI and fluorescently labeled antibodies to the leukocyte marker CD45 and the epithelial cell marker Pdx-1. Using fluorescence microscopy, Pdx1+/DAPI+/CD45- EpCAM captured cells with intact cellular morphology will be counted as CTC’s by a blinded technician. Cells will be stored at -80°C for additional analyses in subsequent grant submissions.
- Fasting insulin will be analyzed with a radioimmunoassay. The intra-assay coefficient of variation is 7%, and sensitivity ranges from 0.137-100.0 ng/mL.[122] We will also assay fasting glucose with an ultraviolet hexokinase assay. The inter-assay coefficient of variation is 2%, and sensitivity ranges from 0.6-45.0mmol/L. The measurement of glucose will serve as a quality control to enable the attribution of elevated fasting levels to insulin resistance, rather than latent T2DM or blood samples that were collected in a non-fasting manner. The measurement of glucose and insulin provides the capacity to quantify insulin resistance using the homeostatic model (HOMA).[123] We will also quantify hemoglobin A1_c_ (HbA1_c_), insulin-Growth Factor-1 (IGF-1), insulin-like growth factor binding protein 3 (IGFBP3), and C-peptide to quantify the metabolic changes in these biomarkers as a result of exercise training.

**Dual Energy X-Ray Absorptiometry (DXA).** DXA imaging is an FDA-approved method to quantify bone mineral density and total body composition including lean muscle mass and fat mass. Novel methodology has been developed to leverage DXA imaging as a technique to quantify VAT. This methodology resulted in a United States Patent (No. 8,483,458), and FDA indication. The Hologic Discovery DXA absorpitometer was validated for measurement of VAT in a cohort of 124 adult men and women, aged 18–90 years.[124] Participants underwent DXA imaging and CT imaging within one-hour of one another in the fasting state. DXA-quantified VAT was strongly correlated with CT-quantified VAT (*r*=0.978 (95% Confidence Interval: 0.968–0.985; *P*<0.0001.[124] DXA-quantified VAT accounted for 95.7% of the variance in CT-quantified VAT (i.e., *R*^2^=0.957).[124] Bland-Altman plots were generated to quantify the magnitude of bias of DXA across a range of VAT areas. The mean bias was +56 cm^3^ and the 95% limits of agreement were –355.0 to +468.0 cm^3^, indicating minimal bias across a range of VAT values.[124] These findings were replicated in a separate sample of 272 African American and Caucasian women.[125] Both studies concluded that the Hologic Discovery DXA absorpitometer, as compared with CT, is a valid and reliable method to quantify VAT.[124,125] DXA offers several unique advantages to CT. First, the cost of DXA is substantially less than that of CT. For this sub-study, DXA scans will be provided free of charge, as part of the NIH-funded Clinical and Translational Research Center (CTRC) Nutritional Core at the University of Pennsylvania (Dr. Zemel is scientific director of the nutritional core which houses the Hologic Discovery scanner, and is a collaborator on this applicatio). Second, CT is frequently indicated for medical procedures in clinical practice. This makes using CT for research challenging given competing clinical care needs. Third, a significant advantage of DXA over CT is that DXA uses less ionizing radiation (0.96 µSV versus 3,100 µSV),[124] making DXA aptly suited for repeated measurements. Patients are reluctant to undergo procedures that require high-doses of ionizing radiation.[126] DXA uniquely circumvents many of the logistical and practical barriers to using CT in a research setting to quantify VAT.

**Pre-Physical Activity Safety Screening.** To maximize participant safety we follow a standardized screening protocol. Prior to completing study procedures, participants are required to obtain written approval to participate in this study from a healthcare provider who is familiar with their health history, such as their primary care physician or medical oncologist. In addition, all participants undergo screening for cardiovascular and other major diseases by means of a health questionnaire (the Physical Activity Readiness Questionnaire (PAR-Q)[127] and other study-specific questions), medication inventory, electrocardiogram (ECG), brief cardiopulmonary exam, and objective assessment of functional exercise capacity using the six-minute walk test (i.e., to identify persons who develop chest pain or shortness of breath during activity).[128] The goal of this series of screening measures is to identify persons who are capable of performing moderate-intensity physical activity without signs and symptoms of cardiac distress. The above information will be reviewed by the study cardiologist who will verify the participant is at acceptable cardiovascular risk to participate in moderate-intensity physical activity. This multi-component screening process has been recommended to minimize the risk of adverse events associated with exercise,[129] and has been successfully implemented in prior randomized clinical trials of physical activity interventions.[130]

**Six-Minute Walk Test** (6MWT). The 6MWT will be performed using American Thoracic Society (ATS) guidelines, including technician training, participant preparation, standardized test conduct, safety, and stopping rules.[128] The goal of the test is to assess the distance the participant can walk in six minutes. Running is not allowed. A specific script is followed to standardize encouragement between participants. A lap counter is used to ensure accuracy of counting the number of times a participant walks the distance between two orange cones set 30 meters apart. Distance is recorded to the nearest meter. The test will take place in the climate controlled setting of the Exercise Medicine Unit. This test has been shown to be reliable (r=0.8 in repeated measures among elders with congestive heart failure),[131] and correlated with physiologic impairments of strength and power in the lower extremity.[132]

**Body weight, height, waist circumference, and sagittal abdominal diameter** will be assessed using a digital scale, scale mounted stadiometer, Gulick tape measure, and abdominal caliper. Height will be assessed at baseline only.

**Diet.** In order to ensure that any effects noted are due to exercise rather than dietary changes, participants will complete a 3-day food record at baseline and follow-up.[135] These food records will be entered and analyzed using Nutrition Coordinating Center software by Penn CTRC registered dietitians trained in its use.

**Accelerometry**. Participants will be asked to wear a 2009 Actigraph GT3X 16 MB and Tractivity triaxial accelerometers for one week before and after the study to objectively assess their activity level.[136] Our research team has experience with objective PA assessment, and the appropriate software needed for analysis of these data.[137-139]

**Self-Reported Questionnaires.** Participants will complete questionnaires relating to demographics,[91,92] alcohol and smoking habits,[140] current medicine and supplement usage.[141] Overall quality of life will be assed using The Functional Assessment of Cancer Therapy-Colorectal (FACT-C),[142] and the SF-36.[143] In addition, participants will complete a series of strategically selected valid and reliable questionnaires to assess issues known to influence the quality of life of people with CC, as acknowledged by the Institute of Medicine and others.[144,145] Pain will be assessed using the Brief Pain Index.[146] Sleep will be assessed using the Pittsburgh Sleep Quality Index.[147] Bowel function will be assessed using the Assessment of Bowel Function Questionnaire.[148] Fatigue will be assessed using the Brief Fatigue Inventory.[149] Neuropathy will be assessed using the Neuropathic Pain Scale.[150] Lastly, participant’s will complete a healthcare utilization and musculoskeletal injury questionnaire at 6-months asking about any healthcare encounters and injuries experienced while in the study that were not identified in the weekly in-person or phone follow-ups, as used in our prior exercise intervention studies among cancer survivors.[93,151]

**Medical record abstraction.** Medical characteristics such as tumor pathology, surgical history, chemotherapy treatment plan, laboratory tests, and any prior comorbid conditions will be extracted from pathology reports and/or the electronic medical record implemented in all practices at the University of Pennsylvania. This data will be collected for all potentially eligible study participants.

**Randomization.** After completion of the eligibility screening and baseline measurements, participants will be randomized evenly to one of three study arms. Randomization will be stratified by stage of cancer (stage II vs. III). Participants will be randomized by the study coordinator using electronic randomization

**Intervention Protocol.** Participants randomized into the low- or high-dose exercise groups will be provided with a treadmill delivered and set up for use in their home and to keep upon study completion (Smooth Fitness 5.65, King of Prussia, PA). Participants randomized to an exercise group will follow the progression described in **Table 1**. This table is a guide, and will be tailored to each participant’s fitness level and physical needs, in acknowledgement that peripheral neuropathy, obesity, and ostomy complication are common clinical issues in this population. In the first 10 weeks of exercise, participants will be required to complete one exercise session in person at the University of Pennsylvania under the supervision of a certified cancer exercise physiologist. A specific exercise intervention laboratory has been set up, with three Smooth Fitness Treadmills, to facilitate these exercise sessions. Parking or subway/bus tokens will be provided to promote adherence. All other exercise will be completed at home with the study-provided treadmill. All exercise sessions will begin with a 5-minute warm up of slow treadmill walking, followed by 30-60 minutes of moderate-intensity walking, followed by a 5-minute cool down of slow treadmill walking. Participants will be discouraged to engage in >60 minutes treadmill walking per day, particularly those in the 300 min∙wk^-1^ group who may seek to engage in 2-3 large doses of exercise per week rather than five or six smaller doses per week. Each week participants will increase their weekly aerobic exercise volume (min∙wk^-1^) by approximately 30 minutes until 150 or 300 min∙wk^-1^ is attained. During supervised exercise sessions, the exercise physiologist will ask participants if they have experienced any health events or are experiencing symptoms or side effects as outlined by the ACSM Guidelines for Exercise Testing and Prescription Table 10.1. Body systems reviewed in Table 10.1 include musculoskeletal, systemic, gastrointestinal, cardiopulmonary, and neurologic as they relate to cancer survivors.[152,153] The oncologist (or Dr. Damjanov) will be consulted for their decision to continue, dose-reduce, or cease exercise as needed.

Table 1. Progression of aerobic exercise

|  | Progression Program of Aerobic Exercise [Minutes per week (min∙wk^-1^)] | | | | | | | | | |
| --- | --- | --- | --- | --- | --- | --- | --- | --- | --- | --- |
| **Group** | Wk 1 | Wk 2 | Wk 3 | Wk 4 | Wk 5 | Wk 6 | Wk 7 | Wk 8 | Wk 9 | Wk10-26 |
| **Low-Dose (150 min∙wk^-1^)** | 30-60 | 60-90 | 90-120 | 120-150 | 150 | 150 | 150 | 150 | 150 | 150 |
| **High-Dose (300 min∙wk^-1^)** | 30-60 | 60-90 | 90-120 | 120-150 | 150-180 | 180-210 | 210-240 | 240-270 | 270-300 | 300 |
| **Control** | Maintain usual activity level → → → → → → → | | | | | | | | | |

**Monitoring of Exercise Compliance.** Each participant will be provided a Polar heart rate monitor (RS400) for the duration of the study. The RS400 heart rate monitor is able to record 99 exercise sessions of data. Each week the exercise physiologist will download the heart rate data onto a secure study laptop to objectively monitor exercise compliance in terms of frequency, duration, and intensity. The exercise compliance information gathered from the heart rate monitors will be reviewed with the participant for positive reinforcement and to develop and tailor the desired dose of exercise for the following week. This data will be used for the overall exercise compliance monitoring.

**Monitoring of Exercise Participation.** If a participant misses a scheduled exercise session, the exercise physiologist will call the participant. All missed exercise sessions will be documented in standardized study participation logs. Participants who demonstrate poor adherence to the study protocol in terms of participation or compliance will be reminded of the study objectives, and discuss any barriers or obstacles to completing the prescribed exercise dose. Each week, participants will be provided with simple, meaningful, attainable, relevant, and timely (SMART) goals to promote exercise self-efficacy, participation, and compliance.[154]

**Control Group.** The control group of this study is a wait-list delayed intervention. Participants randomized into the control group will be asked to maintain their usual levels of PA until the 6 month visit. Participants will be contacted by telephone once per week by the study coordinator to identify and record any incident health events, and to maintain enthusiasm about participation in the study (despite being in wait-list). After completing study measurements at 6-months, control group participants will be provided with a treadmill delivered and set up in their home (to keep), and a 1-month exercise program tailored to their fitness level.

***2. Statistical Analysis***

**Primary Outcomes**

We will randomize 13 participants to each of the three study arms (control, 150 min∙wk^-1^, 300 min∙wk^-1^), to achieve 10 per group who complete the study (76% completion rate). Participants who have a cancer recurrence during the study will be excluded from the primary outcome analysis. The sample size is powered for the primary outcome biomarkers (sICAM-1 and sVICAM-1) with a hypothesis for a linear trend. The standard deviation of sICAM-1 and sVCAM-1 are 30 and 103 units, respectively. Against the hypothesis of a dose-response relationship with decreases of 22 and 44 units for low- and high-dose respectively for sICAM-1 and 74 and 148 units for sVCAM-1, 30 subjects provide 80% power for two, two-sided 0.025 tests (one for sICAM-1 and one for sVCAM-1, with the Type I error adjusted to maintain the experiment-wise error rate of 0.05). These reductions are consistent with prior exercise interventions in non-cancer survivorship populations, where clinically significant reductions in sICAM-1 and sVCAM-1 occur with 30 and 75 unit changes, respectively.[99-103]

The feasibility of exercise will be quantified by comparing the proportion of participants in each group who achieve ≥80% of their prescribed exercise dose. The incidence of exercise dose-alteration will be quantified by comparing the proportion of participants who require a dose-reduction or cessation of exercise in each group. The indication for the dose-reduction or cessation will also be described qualitatively. As this aim intends to describe feasibility, we state no directional hypothesis. For the primary biomarker outcomes, we will use a linear mixed-effects regression to compare the change in biomarker levels for each group while controlling for other variables likely to influence sICAM-1 and sVCAM-1 (in addition to the effects from treatment group). Potential confounders (e.g., change in diet, sleep, or body weight) will be screened using univariate models; those that appear to be associated with the outcome will be entered sequentially in multivariate models. We will initially fit models including dose as a linear term, and assess model fit using standard methods. Alternatives to a linear dose trend include transformations of dose such as average actual dose, log-transformed dose per week, or second-order polynomial (i.e., dose and dose^2^). In the event that a dose-response relationship does not exist, we will compare each of the two intervention groups (low- and high-dose exercise) to the control arm separately. Using a two-sided t-test we will have 80% power to detect a difference in of 32 units for sICAM-1 and 112 units for sVCAM-1, utilizing the sequential testing procedure of Benjamini and Hochberg to protect the family-wise error rate.[155]

**Secondary Outcomes**

The sample size of the COURAGE trial is sufficient to explore the dose-response effects of exercise on VAT and fasting insulin. In the STRRIDE study, changes in VAT among the control, low-dose exercise, and high-dose exercise groups were +8.6%, +1.7%, and –6.9%, respectively.[106-108] Using these point-estimates and a standard deviation of ±17%, we will have 80% power to test the hypothesis of a linear dose-response relationship between exercise and VAT across the three study groups with a type-I error rate of 0.05. In the STRRIDE study, changes in fasting insulin among the control, low-dose exercise, and high-dose exercise groups were +1.1 *μ*U/mL, –0.5 *μ*U/mL, and –0.9 *μ*U/mL, respectively.[114] Using these point-estimates and a standard deviation of ±0.7 *μ*U/mL, we will have ≥80% power to test the hypothesis of a linear dose-response relationship between exercise and fasting insulin across the three study groups with a type-I error rate of 0.05. For our exploratory correlational analysis (i.e., exploratory aim), we will have 80% power to detect a correlation of *≥*0.40 between the exercise-induced improvements in VAT and the exercise-induced improvements in fasting insulin.

For the outcomes of VAT and fasting insulin, we will use a linear mixed-effects regression model to compare the change in outcome levels for each treatment group while simultaneously controlling for other variables identified to influence VAT and fasting insulin (i.e., a multivariable adjusted linear mixed model). Potential confounders (e.g., changes in diet, sleep, and smoking) will be screened using univariate models; those that appear to be associated with the outcome will be entered sequentially in multivariate models. We will fit models including dose as a linear term, and assess model fit using standard methods. Alternatives to a linear dose trend include transformations of dose such as average actual dose, log-transformed dose per week, or second-order polynomial (i.e., dose and dose^2^). In the event that a dose-response relationship does not exist, we will compare each of the two intervention groups (low- and high-dose exercise) to the control arm separately. In our exploratory correlational analyses, we will calculate Pearson’s product-moment correlation coefficient (*r*) to quantify the strength of the relationship between the exercise-induced improvements in VAT and the exercise-induced improvements in fasting insulin.

***3.*** ***Confidentiality***

How will confidentiality of data be maintained? Check all that apply.

Paper-based records will be kept in a secure location and only be accessible to personnel involved in the study.

Computer-based files will only be made available to personnel involved in the study through the use of access privileges and passwords.

Prior to access to any study-related information, personnel will be required to sign statements agreeing to protect the security and confidentiality of identifiable information.

Whenever feasible, identifiers will be removed from study-related information.

A Certificate of Confidentiality will be obtained, because the research could place the subject at risk of criminal or civil liability or cause damage to the subject’s financial standing, employability, or liability.

A waiver of documentation of consent is being requested, because the only link between the subject and the study would be the consent document and the primary risk is a breach of confidentiality. (This is not an option for FDA-regulated research.)

Precautions are in place to ensure the data is secure by using passwords and encryption, because the research involves web-based surveys.

Audio and/or video recordings will be transcribed and then destroyed to eliminate audible identification of subjects.

Other *(specify):*

Data will be de-identified upon completion of all study activities, including any resulting publications. Data will be kept confidential throughout the course of the project by ensuring that the staff in position of or with access to the data will have completed all appropriate training and will keep paper files in a locked cabinet and electronic files will be in password protected files behind a firewall. Data will be physically stored in a locked file cabinet in an office at Penn. No data will become part of the patient’s permanent record. The data will be kept for a minimum of five years after study completion.

***4. Privacy***

We will keep all patient databases private. All measurements will be taken in a private room. Participants will be told in advance that if they are selected into one of the exercise groups, they may be in a group setting with other CC survivors and it will not be possible to conceal the fact that they are CC survivors from other participants. For example, after randomization if two participants opt to attend their supervised exercise sessions at the same time. We will remind the participants to respect privacy by not revealing the identity of their fellow group members without permission to do so.

***HIPAA***

Describe if data to be collected contain any protected health information (PHI) and specify which PHI is to be collected. Health information is determined to be PHI if it contains any of the following identifiers:

| - Name, address, telephone number, date of birth - Personal and family medical history - Current and past medications or therapies - Hospitalization & other institutional care information | - Information from a physical examination that generally also includes blood pressure reading, heart rate, breathing rate and temperature - Results of tests and procedures you will undergo during this research study as described in the informed consent form. |  |
| --- | --- | --- |

Data will be stored in locked file cabinets and in password protected files behind firewalls. Only staff with appropriate training to ensure that privacy is protected according to HIPAA and human subjects’ protection programs will be allowed access to participant data.

***5. Tissue Specimens***

Blood will be obtained at baseline and six-months.

The specimens:

- Were not collected during regular clinical care
- Would not have otherwise been discarded
- Are not publicly available
- Are collected as part of the research protocol
- This research involves banking of blood for future uses

***6. Genetic Testing***

Not applicable

**RISK/BENEFIT ASSESSMENT**

***1. Potential Study Risks***

**Likely (greater than 20%)**

- Muscle or joint soreness or injury from the maximal exercise test or exercise training. These are usually mild, last a week or less, and require little medical attention, if any. The types of injuries that are most common from aerobic exercise such as what you will do in the exercise test or training are hip, knee, ankle, or foot joint pain, or muscle aches. Sometimes these injuries are mild enough that you will not need to seek any medical attention and your daily activities will not be altered. It is estimated that over any given month, 4% of adults who walk for exercise on a regular basis will incur an injury severe enough to cause a change in activities of daily living for a week or more and that require medical attention. These injuries are rarely serious in nature and generally resolve with reduced activity and greater attention to appropriate footwear, gradual increase in training intensity, and adequate stretching.
- Mild abrasions and stinging at the site where we clean the skin for the electrodes for the treadmill test.
- DEXA scan risks:
  - You will receive a radiation dose. This radiation dose is not necessary for your medical care and will occur only as a result of your participation in the study.
  - At doses much higher than you will receive, radiation is known to increase the risk of developing cancer after many years. At the doses you will receive, it is very likely that you will see no effects at all.

**Less Likely (Less than 20%)**

- The potential for embarrassment at having weight measured or answering specific survey questions
- There is a small risk of infection when your blood is being taken, but the risk is minimal as all needles and equipment are sterilized and the procedures are performed by a trained professional. You may experience some mild to moderate pain lasting a few seconds upon insertion of the needle used to draw the blood. You may also get a bruise from the blood draw.
- Some personal health information could be revealed, despite efforts to avoid this.

**Serious but rare (Less than 2–3%)**

- 6-minute walk test risks. The risk of cardiovascular events from the six minute walk test is less than 1 in 5000. This test will be performed within the Exercise Medicine Unit at the Presbyterian Hospital location of the Clinical and Translational Research Center. Individuals for whom participating in this test is unsafe will be excluded from participation in the study.

***2. Potential Study Benefits***

**Potential Benefits to Subjects:** Participants in the exercise groups are expected to experience improved cardiorespiratory function, and improved quality of life. All participants will receive a treadmill delivered and set up in their home for no charge to them. Participants randomized to the exercise group will receive their treadmill at the beginning of the study. Participants randomized to the control group will receive their treadmill after completing the study. There may be benefits to their cell adhesion and circulating tumor cell function as well.

**Potential Benefits to Others:** Regardless of whether the hypotheses regarding the mechanisms of colon cancer recurrence are supported, this work will improve our knowledge of the feasibility, safety, and the benefits of two distinct doses of aerobic exercise on quality of life and functional well-being among colon cancer survivors.

**Importance of the Knowledge to be Gained:** If the primary hypothesis of this study is supported, this will support the further exploration of the dose response effects of exercise for reducing the risk of recurrence in colon cancer. If the secondary hypotheses are supported, this trial will increase confidence in the potential for exercise to improve long-term outcomes among colon cancer including cancer-specific and overall survival. If our hypotheses are not supported, this study will improve our knowledge about exercise prescription in this population. Upon study completion, the investigators will summarize the major findings of the study, such as feasibility of exercise, and notify all study subjects via a mailed letter. The purpose of this letter will be to thank study subjects for their participation and to inform them of the findings of the study.

***3. Alternatives to Participation***

The participants recruited would have the option of paying for a gym membership at a facility with fitness professionals who are trained in teaching a progressive aerobic exercise protocol for CC cancer survivors.

***4. Data and Safety Monitoring***

**Introduction**

Since this study constitutes a clinical trial, although we are not study experimental drugs, the intervention is experimental with the attendant risks described above in the Human Subjects section. Therefore it is appropriate to have a Data and Safety Monitoring Plan (DSMP) to monitor the study with an attendant monitoring plan as outlined below.

Every effort will be made to report an event as a diagnosis, not as a list of symptoms. Symptoms that led to the diagnosis should be included in the event description, but should not be the actual event.

Once an event is reported, you must keep the information accurate and current in Velos. If new/updated information is learned about the event, the event should be amended or corrected promptly.

Only an investigator may determine the grade, attribution and expectedness.

NOTE: The DSMC reserves the right to modify the reporting requirements for studies of specific interest.

On-Site subjects (this includes any subjects enrolled at other sites on an in-house study)

1. All grade 3 or higher events (AE or SAE) within five business days of knowledge.
2. All unexpected deaths within 24 hours of knowledge.
3. All others deaths within 30 days of knowledge. Deaths of subjects off-study for greater than 30 days from the last study treatment/intervention are not reportable with the following exceptions:
4. Deaths on in-house gene or cellular-therapies
5. Deaths on in-house studies utilizing on-campus manufacturing of the study agent(s) or components of the study agent(s)
6. Deaths on first-in-human studies

Off-site (external sponsors)

1. All SAEs (on the specific protocol opened here only) defined as reportable in the protocol for Phase I and II studies.
2. SAEs on cooperative or industry sponsored Phase III studies do not have to be reported.
3. SAEs on consortium or foundation sponsored (not just funded) Phase III have to be reported if the protocol defines them as reportable.
4. All deaths on first-in-human studies, must be reported at the time the event is reported to the sponsor.

We will establish a comprehensive plan to monitor the data and safety of the human participants in this trial. The PI, Dr. Schmitz, will ultimately be responsible for ongoing on continuous monitoring of the safety of the human participants in the trial, as guided by the DSMP. The proposed study aims to determine the effects of varying doses of moderate-intensity aerobic exercise on various biomarkers among a clinical population with colon cancer. The intervention protocol poses minimal risks to participants. Because of this risk status, the DSMP for this trial focuses on monitoring by the principal investigator and biostatistician, along with prompt reporting of excessive adverse events and any serious adverse events to the NIH, the IRB, the University of Pennsylvania’s Abramson Cancer Center Clinical Trials Scientific Review and Monitoring Committee (CTSRMC).

Who will monitor this study? Check all that apply.

Principal Investigator

Sponsor or contract research organization

NCI sponsored cooperative group

Cancer Center (if mandated by CTSMRC)

Medical monitor

Safety monitoring committee

Data and safety monitoring board

**DSMP reports**

DSMP reports will be produced by the study coordinator. We will adapt adverse event reporting forms from our prior ongoing and completed study for this study. Safety reports will be sent to the study statistician and the PI. The project coordinator will be responsible for assembling the data and producing these reports, as well as assuring that all parties obtain copies of these reports. The frequency of data review for this study is summarized below:

| Data Type | Frequency of Review |
| --- | --- |
| Subject accrual (adherence to protocol regarding demographics, inclusion/exclusion) | Quarterly |
| Adverse event rates (injuries, symptom changes requiring altered exercise prescription) | Quarterly |
| Compliance to treatment, dropout rates | Monthly |

**Responsibilities of the PI and Statistician**

The PI and statistician will meet once before subjects are accrued into the trial to review safety protocols and stopping rules and ensure that the procedures for stopping the trial early are clear and empirically based. Thereafter, the PI and statistician will meet quarterly to review subject accrual, adverse event rates, stopping rules report, and compliance to the intervention. If there is any interim concern between these meetings, they will be handled via email as much as possible. A checklist will be developed and used to determine whether any corrective action is needed at any particular time during the study. The PI and statistician will be allowed to trigger an ad hoc review at any time. Stopping rule violations will be avoided by these actions, but if they should occur, they will be communicated to the NIH and to the IRB. In addition, the PI and statistician may comment on whether the study investigator needs to report any specific out of range measurement results to a participant and/or his/her physician.

**Measurement and reporting of subject accrual, adherence to inclusion/exclusion criteria**

Review of the rate of accrual, and adherence to inclusion and exclusion criteria will occur quarterly during the recruitment phase. Review to assure that participants meet eligibility criteria and ethnic diversity goals outlined in the grant proposal will occur quarterly during the recruitment phase.

**Measurement and reporting of adverse events**

Adverse events will be collected as they occur. Musculoskeletal injuries and previously undocumented major medial events incurred during the intervention will be systematically collected on all participants at the final six month study visit using standardized forms from our prior work. The adverse event form will be used by study staff to report injuries or other adverse events reported by participants throughout the study.

**Stopping rules**

**Stopping rules regarding injuries from exercise training**

There is some level of injury expected from exercise training. The PI and study statistician will review exercise training injury rates quarterly. If the rate of injuries attributed to exercise and lasting a week or longer is more than 4-fold higher over 3 months among treatment than control group participants, it will be reported to the NIH, the IRB, the University of Pennsylvania’s Abramson Cancer Center Clinical Trials Scientific Review and Monitoring Committee (CTSRMC). Most exercise training injuries are mild in nature, and allow a complete return to the same activities after slowing reintroducing the exercise that caused the injury. Another approach is to substitute a different mode of exercise that does not place excessive demand on injured muscle, tendon, ligament, or other soft tissue fibers.

**Measurement and reporting of participant compliance to treatment protocol**

Once participants are randomized to the treatment groups and being the intervention, data on adherence to exercise sessions will be reviewed weekly by the clinical exercise physiologist, and reviewed monthly by the PI and study statistician. If the PI or statistician has concerns about whether adherence has reached a level that might inhibit the ability of the study to test its primary hypothesis, methods for improving adherence will be discussed with intervention staff.

**Limits of Assumptions**

It is possible that baseline differences between groups, excessive attrition and/or missing data by the interim measurement time points will limit the value of data analysis of measurements. Baseline differences will be evaluated after the first measurement time points and effects on the power to detect differences in the primary outcomes will be evaluated and communicated to the biostatistician and the NIH. We also assume 80% of randomized participants will complete the study.

**Limits of Rules**

We acknowledge that there are other situations that could occur that might warrant stopping the trial and will have a section on the safety report entitled: “Other situations that have occurred since the last safety report that warrant discussion” to allow for communication of concerns to the study PI and biostatistician.

**Reporting of Deviations & Exceptions**

In order to harmonize with the IRB, the DSMC has changed its designations from Deviations and Violations to Exceptions and Deviations.

Study Exceptions the DSMC will not Approve:

Exceptions to eligibility, treatment/dosing, contraindicated treatment/therapies/interventions or safety tests will not be approved for any of the following types of studies:

1. Any investigator-initiated treatment study.
2. Any investigator-initiated study utilizing an intervention with therapeutic intent.
3. Any Phase III study, regardless of sponsor (in-house, cooperative group, industry, consortium, etc.).
4. Any study involving on-campus manufacturing of any component, regardless of sponsor. 5. Any first in-human study.

Requests that fall into any of the above categories will receive an automatic rejection "The DSMC has rejected this request."

***Exception***

A one time, intentional action or process that departs from the IRB and CTSRMC approved study protocol, intended for one occurrence. If the action disrupts the study progress, such that the study design or outcome (endpoints) may be compromised, or the action compromises the safety and welfare of study subjects, advance documented IRB and DSMC approval is required.

• For exceptions on Industry or Cooperative group sponsored protocols, written approval must be obtained from the Sponsor prior to submitting your exception request to the DSMC.

- For in-house studies with a Medical Monitor or Safety Monitoring Committee (not DSMB), approval must be obtained from the Medical Monitor or Safety Monitoring Committee prior to submitting your exception request to the DSMC.

***Deviation***

A one time, unintentional action or process that departs from the IRB and DSMC approved study protocol, involving one incident and identified retrospectively, after the event occurred. If the impact on the protocol disrupts the study design, may affect the outcome (endpoints) or compromises the safety and welfare of the subjects, the deviation must be reported to the DSMC within 5 business days and the IRB within 10 business days.

The ACC Department of Compliance and Monitoring (DOCM) has created two form that have also been reviewed by the IRB. These forms allow you to both document your exceptions/deviations and the assessment of whether or not the issue is reportable. You are not required to use these forms, rather, we recognize that many researchers/groups do not have a standardized process to document compliance with IRB and DSMC requirements, thus, if you don't already have a process, or are looking for another process, we encourage you to use the attached optional forms found at: [www.cstrmc.org/study_deviation.php](http://www.cstrmc.org/study_deviation.php).

***5. Management of Information for Multi-center Research where a Penn Investigator is the Lead Investigator of a multi- center study, or Penn is the lead site in a multi-site study.***

This is a single center trial.

***6. Risk/Benefit Assessment***

Once participants are randomized, a certified clinical exercise physiologist will review their current exercise habits and baseline fitness levels and develop a plan to gradually increase the minutes of exercise each week to the prescribed dose. The intensity of their aerobic exercise will be objectively monitored and recorded using heart rate monitors. The certified clinical exercise physiologist will review the objective recording produced by the heart rate monitor each week, and ask the participant if they are experiencing any symptoms that would preclude an increase or maintenance of the currently prescribed dose of exercise. The participants medical care team will be informed of symptom changes and consulted regarding whether is it appropriate to dose-reduce the exercise or cease exercise training all together. In our prior exercise study, the Schmitz research laboratory suggests 20% of participants will require exercise dose modifications during the 6 month intervention. Participants who are found to have recurrent cancer will be allowed to continue participation in the study, unless their medical oncologist or other physician asks them to stop or reduce their level of exercise. Participants who are found to have a recurrence will be excluded in data analysis. Study staff will work closely with the medical team to ensure the participant receives appropriate medical and psychosocial care and follow-up as necessary.

**SUBJECT COMPENSATION**

All study participants will receive a Smooth Fitness 5.65 treadmill valued at $999.00. The treadmill will be delivered to and setup in participant homes. Participants in the exercise groups will receive their treadmill after randomization to use for exercise. Participants in the control group will receive their treadmill after completing their six-month post-intervention measurements.

**INFORMED CONSENT**

***1. Consent Process***

The Informed consent process will take place individually or in groups in an enclosed conference room. The study purpose and all study procedures, risks, and benefits, will be outlined for potential participants using simple understandable (eighth grade level) language. The fact that the research is voluntary and that participants may withdraw at any time without fear that this would negatively affect future medical care will be emphasized. The potential participants will be asked if they have any questions and to repeat back to the research staff member the purpose of the study, the procedures, risks, benefits, and the fact that participation is voluntary before being allowed to sign the consent form. Participants will not be required to sign their informed consent during the informed consent session. All participants will be encouraged to ask any questions they may have prior to deciding if they chose to enroll in the study. Based on the inclusion criteria (described in earlier sections of this protocol), we anticipate all study participants will be competent to provide their informed consent if they chose to do so.

***2. Waiver of Authorization***

No waiver of authorization is requested.

**RESOURCES NECESSARY FOR HUMAN RESEARCH PROTECTION**

Kathryn H. Schmitz, PhD, MPH, Principal Investigator, Exercise Physiologist, Epidemiologist

Nevena Damjanov, MD, Co-Investigator, Medical Oncologist

Andrea Troxel, ScD, Co-Investigator, Biostatistician

Andrew Rhim, MD, Co-Investigator, Gastroenterologist

Bonnie Ky, MD, MSCE, Co-Investigator, Cardiologist

Michael Rickels, MD, MS, Co-Investigator, Endocrinologist

Babette Zemel, PhD, Co-Investigator, Anthropologist

Anil Rustgi, MD, Significant Contributor, Gastroenterologist

Justin C. Brown, MA, Study Coordinator

Each of the staff will have completed all required human subjects and HIPAA training prior to interacting with any participants or data. Prior to initiating any study activities a meeting will be held at which all of these staff/faculty will be gathered to review the complete protocol and ensure that all who are working on this project understand the full scope of what the participants are asked to do.

**REFERENCES**

**1. Siegel R, Desantis C, Virgo K, Stein K, Mariotto A, Smith T, et al. Cancer treatment and survivorship statistics, 2012. CA Cancer J Clin 2012; 62:220-241.**

**2. Chang GJ, Rodriguez-Bigas MA, Skibber JM, Moyer VA Lymph node evaluation and survival after curative resection of colon cancer: systematic review. J Natl Cancer Inst 2007; 99:433-441.**

**3. Jessup JM, Stewart A, Greene FL, Minsky BD Adjuvant chemotherapy for stage III colon cancer: implications of race/ethnicity, age, and differentiation. JAMA 2005; 294:2703-2711.**

**4. Benson AB,3rd, Schrag D, Somerfield MR, Cohen AM, Figueredo AT, Flynn PJ, et al. American Society of Clinical Oncology recommendations on adjuvant chemotherapy for stage II colon cancer. J Clin Oncol 2004; 22:3408-3419.**

**5. Lacy AM, Garcia-Valdecasas JC, Delgado S, Castells A, Taura P, Pique JM, et al. Laparoscopy-assisted colectomy versus open colectomy for treatment of non-metastatic colon cancer: a randomised trial. Lancet 2002; 359:2224-2229.**

**6. Sargent DJ, Wieand HS, Haller DG, Gray R, Benedetti JK, Buyse M, et al. Disease-free survival versus overall survival as a primary end point for adjuvant colon cancer studies: individual patient data from 20,898 patients on 18 randomized trials. J Clin Oncol 2005; 23:8664-8670.**

**7. Makrilia N, Kollias A, Manolopoulos L, Syrigos K Cell adhesion molecules: role and clinical significance in cancer. Cancer Invest 2009; 27:1023-1037.**

**8. Gupta GP, Massague J Cancer metastasis: building a framework. Cell 2006; 127:679-695.**

**9. Chambers AF, Groom AC, MacDonald IC Dissemination and growth of cancer cells in metastatic sites. Nat Rev Cancer 2002; 2:563-572.**

**10. Rahbari NN, Aigner M, Thorlund K, Mollberg N, Motschall E, Jensen K, et al. Meta-analysis shows that detection of circulating tumor cells indicates poor prognosis in patients with colorectal cancer. Gastroenterology 2010; 138:1714-1726.**

**11. Bosch B, Guller U, Schnider A, Maurer R, Harder F, Metzger U, et al. Perioperative detection of disseminated tumour cells is an independent prognostic factor in patients with colorectal cancer. Br J Surg 2003; 90:882-888.**

**12. Uen YH, Lu CY, Tsai HL, Yu FJ, Huang MY, Cheng TL, et al. Persistent presence of postoperative circulating tumor cells is a poor prognostic factor for patients with stage I-III colorectal cancer after curative resection. Ann Surg Oncol 2008; 15:2120-2128.**

**13. Yie SM, Lou B, Ye SR, Cao M, He X, Li P, et al. Detection of survivin-expressing circulating cancer cells (CCCs) in peripheral blood of patients with gastric and colorectal cancer reveals high risks of relapse. Ann Surg Oncol 2008; 15:3073-3082.**

**14. Cohen SJ, Punt CJ, Iannotti N, Saidman BH, Sabbath KD, Gabrail NY, et al. Relationship of circulating tumor cells to tumor response, progression-free survival, and overall survival in patients with metastatic colorectal cancer. J Clin Oncol 2008; 26:3213-3221.**

**15. Iinuma H, Watanabe T, Mimori K, Adachi M, Hayashi N, Tamura J, et al. Clinical significance of circulating tumor cells, including cancer stem-like cells, in peripheral blood for recurrence and prognosis in patients with Dukes' stage B and C colorectal cancer. J Clin Oncol 2011; 29:1547-1555.**

**16. Hardingham JE, Kotasek D, Sage RE, Eaton MC, Pascoe VH, Dobrovic A Detection of circulating tumor cells in colorectal cancer by immunobead-PCR is a sensitive prognostic marker for relapse of disease. Mol Med 1995; 1:789-794.**

**17. Parkinson DR, Dracopoli N, Petty BG, Compton C, Cristofanilli M, Deisseroth A, et al. Considerations in the development of circulating tumor cell technology for clinical use. J Transl Med 2012; 10:138-5876-10-138.**

**18. Riethdorf S, Fritsche H, Muller V, Rau T, Schindlbeck C, Rack B, et al. Detection of circulating tumor cells in peripheral blood of patients with metastatic breast cancer: a validation study of the CellSearch system. Clin Cancer Res 2007; 13:920-928.**

**19. Miller MC, Doyle GV, Terstappen LW Significance of circulating tumor cells detected by the CellSearch system in patients with metastatic breast colorectal and prostate cancer. Journal of oncology 2009; 2010.**

**20. Kirby BJ, Jodari M, Loftus MS, Gakhar G, Pratt ED, Chanel-Vos C, et al. Functional characterization of circulating tumor cells with a prostate-cancer-specific microfluidic device. PLoS One 2012; 7:e35976.**

**21. Rhim AD, Mirek ET, Aiello NM, Maitra A, Bailey JM, McAllister F, et al. EMT and dissemination precede pancreatic tumor formation. Cell 2012; 148:349-361.**

**22. Pantel K, Denève E, Nocca D, Coffy A, Vendrell J, Maudelonde T, et al. Circulating epithelial cells in patients with benign colon diseases. Clin Chem 2012; 58:936-940.**

**23. Giovannucci E Insulin, insulin-like growth factors and colon cancer: a review of the evidence. J Nutr 2001; 131:3109S-3120S.**

**24. Meyerhardt JA, Heseltine D, Niedzwiecki D, Hollis D, Saltz LB, Mayer RJ, et al. Impact of physical activity on cancer recurrence and survival in patients with stage III colon cancer: findings from CALGB 89803. J Clin Oncol 2006; 24:3535-3541.**

**25. Meyerhardt JA, Giovannucci EL, Holmes MD, Chan AT, Chan JA, Colditz GA, et al. Physical activity and survival after colorectal cancer diagnosis. J Clin Oncol 2006; 24:3527-3534.**

**26. Meyerhardt JA, Giovannucci EL, Ogino S, Kirkner GJ, Chan AT, Willett W, et al. Physical activity and male colorectal cancer survival. Arch Intern Med 2009; 169:2102-2108.**

**27. Alexiou D, Karayiannakis AJ, Syrigos KN, Zbar A, Kremmyda A, Bramis I, et al. Serum levels of E-selectin, ICAM-1 and VCAM-1 in colorectal cancer patients: correlations with clinicopathological features, patient survival and tumour surgery. Eur J Cancer 2001; 37:2392-2397.**

**28. Dymicka-Piekarska V, Kemona H Does colorectal cancer clinical advancement affect adhesion molecules (sP-selectin, sE-selectin and ICAM-1) concentration? Thromb Res 2009; 124:80-83.**

**29. Cavallaro U, Dejana E Adhesion molecule signalling: not always a sticky business. Nat Rev Mol Cell Biol 2011; 12:189-197.**

**30. Cavallaro U, Christofori G Cell adhesion and signalling by cadherins and Ig-CAMs in cancer. Nat Rev Cancer 2004; 4:118-132.**

**31. Paschos KA, Canovas D, Bird NC The role of cell adhesion molecules in the progression of colorectal cancer and the development of liver metastasis. Cell Signal 2009; 21:665-674.**

**32. Velikova G, Banks RE, Gearing A, Hemingway I, Forbes MA, Preston SR, et al. Serum concentrations of soluble adhesion molecules in patients with colorectal cancer. Br J Cancer 1998; 77:1857-1863.**

**33. Binion DG, Heidemann J, Li MS, Nelson VM, Otterson MF, Rafiee P Vascular cell adhesion molecule-1 expression in human intestinal microvascular endothelial cells is regulated by PI 3-kinase/Akt/MAPK/NF-kappaB: inhibitory role of curcumin. Am J Physiol Gastrointest Liver Physiol 2009; 297:G259-68.**

**34. Niessen HW, Krijnen PA, Visser CA, Meijer CJ, Hack CE Intercellular adhesion molecule-1 in the heart. Ann N Y Acad Sci 2002; 973:573-585.**

**35. Wang H, Quah SY, Dong JM, Manser E, Tang JP, Zeng Q PRL-3 down-regulates PTEN expression and signals through PI3K to promote epithelial-mesenchymal transition. Cancer Res 2007; 67:2922-2926.**

**36. Sordat I, Decraene C, Silvestre T, Petermann O, Auffray C, Pietu G, et al. Complementary DNA arrays identify CD63 tetraspanin and alpha3 integrin chain as differentially expressed in low and high metastatic human colon carcinoma cells. Lab Invest 2002; 82:1715-1724.**

**37. Karin M, Greten FR NF-kappaB: linking inflammation and immunity to cancer development and progression. Nat Rev Immunol 2005; 5:749-759.**

**38. Agrez MV Cell adhesion molecules and colon cancer. Aust N Z J Surg 1996; 66:791-798.**

**39. Toiyama Y, Miki C, Inoue Y, Okugawa Y, Koike Y, Yokoe T, et al. Soluble intercellular adhesion molecule-1 as a prognostic marker for stage II colorectal cancer patients. Ann Surg Oncol 2008; 15:1617-1624.**

**40. Giannoulis K, Angouridaki C, Fountzilas G, Papapolychroniadis C, Giannoulis E, Gamvros O Serum concentrations of soluble ICAM-1 and VCAM-1 in patients with colorectal cancer. Clinical implications. Tech Coloproctol 2004; 8 Suppl 1:s65-7.**

**41. Okugawa Y, Miki C, Toiyama Y, Koike Y, Inoue Y, Kusunoki M Serum level of soluble vascular cell adhesion molecule 1 is a valuable prognostic marker in colorectal carcinoma. Dis Colon Rectum 2009; 52:1330-1336.**

**42. Shantha Kumara HM, Tohme ST, Herath SA, Yan X, Senagore AJ, Nasar A, et al. Plasma soluble vascular adhesion molecule-1 levels are persistently elevated during the first month after colorectal cancer resection. Surg Endosc 2012; 26:1759-1764.**

**43. Raa ST, Oosterling SJ, van der Kaaij NP, van den Tol MP, Beelen RH, Meijer S, et al. Surgery promotes implantation of disseminated tumor cells, but does not increase growth of tumor cell clusters. J Surg Oncol 2005; 92:124-129.**

**44. Yamada Y, Arao T, Matsumoto K, Gupta V, Tan W, Fedynyshyn J, et al. Plasma concentrations of VCAM-1 and PAI-1: a predictive biomarker for post-operative recurrence in colorectal cancer. Cancer Sci 2010; 101:1886-1890.**

**45. Toiyama Y, Miki C, Inoue Y, Kawamoto A, Kusunoki M Circulating form of human vascular adhesion protein-1 (VAP-1): decreased serum levels in progression of colorectal cancer and predictive marker of lymphatic and hepatic metastasis. J Surg Oncol 2009; 99:368-372.**

**46. Roselli M, Guadagni F, Martini F, Spila A, Mariotti S, D'Alessandro R, et al. Association between serum carcinoembryonic antigen and endothelial cell adhesion molecules in colorectal cancer. Oncology 2003; 65:132-138.**

**47. Giavazzi R, Chirivi RG, Garofalo A, Rambaldi A, Hemingway I, Pigott R, et al. Soluble intercellular adhesion molecule 1 is released by human melanoma cells and is associated with tumor growth in nude mice. Cancer Res 1992; 52:2628-2630.**

**48. Gho YS, Kim PN, Li H, Elkin M, Kleinman HK Stimulation of tumor growth by human soluble intercellular adhesion molecule-1. Cancer Res 2001; 61:4253-4257.**

**49. Kelly C, O'keane J, Orellana J, Schroy P, Yang S, LaMont J, et al. Human colon cancer cells express ICAM-1 in vivo and support LFA-1-dependent lymphocyte adhesion in vitro. American Journal of Physiology-Gastrointestinal and Liver Physiology 1992; 263:G864-G870.**

**50. Shuster A, Patlas M, Pinthus JH, Mourtzakis M The clinical importance of visceral adiposity: a critical review of methods for visceral adipose tissue analysis. Br J Radiol 2012; 85:1-10.**

**51. Ibrahim MM Subcutaneous and visceral adipose tissue: structural and functional differences. Obesity reviews 2010; 11:11-18.**

**52. Tchernof A, Despres JP Pathophysiology of human visceral obesity: an update. Physiol Rev 2013; 93:359-404.**

**53. Balistreri CR, Caruso C, Candore G The role of adipose tissue and adipokines in obesity-related inflammatory diseases. Mediators Inflamm 2010; 2010:802078.**

**54. Ahima RS, Flier JS Adipose tissue as an endocrine organ. Trends in Endocrinology & Metabolism 2000; 11:327-332.**

**55. Doyle SL, Donohoe CL, Lysaght J, Reynolds JV Symposium 3: Obesity-related cancers Visceral obesity, metabolic syndrome, insulin resistance and cancer. survival 2012; 38:41.**

**56. Rickles AS, Iannuzzi JC, Kelly KN, Garimella V, Fleming FJ, Monson JR The relationship between visceral obesity and colorectal cancer. Colorectal Cancer 2013; 2:265-275.**

**57. Rickles AS, Iannuzzi JC, Mironov O, Deeb A, Sharma A, Fleming FJ, et al. Visceral obesity and colorectal cancer: are we missing the boat with BMI? Journal of Gastrointestinal Surgery 2013; 17:133-143.**

**58. Moon HG, Ju YT, Jeong CY, Jung EJ, Lee YJ, Hong SC, et al. Visceral obesity may affect oncologic outcome in patients with colorectal cancer. Ann Surg Oncol 2008; 15:1918-1922.**

**59. Guiu B, Petit JM, Bonnetain F, Ladoire S, Guiu S, Cercueil J, et al. Visceral fat area is an independent predictive biomarker of outcome after first-line bevacizumab-based treatment in metastatic colorectal cancer. Gut 2010; 59:341-347.**

**60. Ballian N, Lubner MG, Munoz A, Harms BA, Heise CP, Foley EF, et al. Visceral obesity is associated with outcomes of total mesorectal excision for rectal adenocarcinoma. J Surg Oncol 2012; 105:365-370.**

**61. Haydon AM, Macinnis RJ, English DR, Giles GG Effect of physical activity and body size on survival after diagnosis with colorectal cancer. Gut 2006; 55:62-67.**

**62. Prizment AE, Flood A, Anderson KE, Folsom AR Survival of women with colon cancer in relation to precancer anthropometric characteristics: the Iowa Women's Health Study. Cancer Epidemiology Biomarkers & Prevention 2010; 19:2229-2237.**

**63. Katzmarzyk P, Mire E, Bouchard C Abdominal obesity and mortality: The Pennington Center Longitudinal Study. Nutrition & diabetes 2012; 2:e42.**

**64. Kuk JL, Katzmarzyk PT, Nichaman MZ, Church TS, Blair SN, Ross R Visceral Fat Is an Independent Predictor of All‐cause Mortality in Men. Obesity 2006; 14:336-341.**

**65. Larsson SC, Wolk A Obesity and colon and rectal cancer risk: a meta-analysis of prospective studies. Am J Clin Nutr 2007; 86:556-565.**

**66. Vrieling A, Kampman E The role of body mass index, physical activity, and diet in colorectal cancer recurrence and survival: a review of the literature. Am J Clin Nutr 2010; 92:471-490.**

**67. Meyerhardt JA, Niedzwiecki D, Hollis D, Saltz LB, Mayer RJ, Nelson H, et al. Impact of body mass index and weight change after treatment on cancer recurrence and survival in patients with stage III colon cancer: findings from Cancer and Leukemia Group B 89803. J Clin Oncol 2008; 26:4109-4115.**

**68. Després J Excess Visceral Adipose Tissue/Ectopic FatThe Missing Link in the Obesity Paradox? J Am Coll Cardiol 2011; 57:1887-1889.**

**69. Jiang B, Zhang X, Du L, Wang Y, Liu D, Han C, et al. Possible roles of insulin, IGF-1 and IGFBPs in initiation and progression of colorectal cancer. World journal of gastroenterology: WJG 2014; 20:1608.**

**70. Sandhu MS, Dunger DB, Giovannucci EL Insulin, insulin-like growth factor-I (IGF-I), IGF binding proteins, their biologic interactions, and colorectal cancer. J Natl Cancer Inst 2002; 94:972-980.**

**71. Seidell JC, Björntorp P, Sjöström L, Kvist H, Sannerstedt R Visceral fat accumulation in men is positively associated with insulin, glucose, and C-peptide levels, but negatively with testosterone levels. Metab Clin Exp 1990; 39:897-901.**

**72. Colman E, Toth M, Katzel L, Fonong T, Gardner A, Poehlman E Body fatness and waist circumference are independent predictors of the age-associated increase in fasting insulin levels in healthy men and women. International journal of obesity and related metabolic disorders: journal of the International Association for the Study of Obesity 1995; 19:798-803.**

**73. Goodpaster BH, Krishnaswami S, Resnick H, Kelley DE, Haggerty C, Harris TB, et al. Association between regional adipose tissue distribution and both type 2 diabetes and impaired glucose tolerance in elderly men and women. Diabetes Care 2003; 26:372-379.**

**74. Racette SB, Evans EM, Weiss EP, Hagberg JM, Holloszy JO Abdominal adiposity is a stronger predictor of insulin resistance than fitness among 50–95 year olds. Diabetes Care 2006; 29:673-678.**

**75. Meyerhardt JA, Catalano PJ, Haller DG, Mayer RJ, Macdonald JS, Benson AB,3rd, et al. Impact of diabetes mellitus on outcomes in patients with colon cancer. J Clin Oncol 2003; 21:433-440.**

**76. Jeon JY, Jeong DH, Park MG, Lee J, Chu SH, Park J, et al. Impact of diabetes on oncologic outcome of colorectal cancer patients: colon vs. rectal cancer. PloS one 2013; 8:e55196.**

**77. Dehal AN, Newton CC, Jacobs EJ, Patel AV, Gapstur SM, Campbell PT Impact of diabetes mellitus and insulin use on survival after colorectal cancer diagnosis: the Cancer Prevention Study-II Nutrition Cohort. J Clin Oncol 2012; 30:53-59.**

**78. Luo J, Lin H, He K, Hendryx M Diabetes and prognosis in older persons with colorectal cancer. Br J Cancer 2014.**

**79. Wolpin BM, Meyerhardt JA, Chan AT, Ng K, Chan JA, Wu K, et al. Insulin, the insulin-like growth factor axis, and mortality in patients with nonmetastatic colorectal cancer. Journal of Clinical Oncology 2009; 27:176-185.**

**80. Haydon AM, Macinnis RJ, English DR, Morris H, Giles GG Physical activity, insulin-like growth factor 1, insulin-like growth factor binding protein 3, and survival from colorectal cancer. Gut 2006; 55:689-694.**

**81. Courneya KS, Booth CM, Gill S, O'Brien P, Vardy J, Friedenreich CM, et al. The Colon Health and Life-Long Exercise Change trial: a randomized trial of the National Cancer Institute of Canada Clinical Trials Group. Curr Oncol 2008; 15:279-285.**

**82. Meyerhardt J Exercise and Metformin in Colorectal Cancer Survivors. . Available from:** [**http://www.clinicaltrials.gov/ct2/show/NCT01340300?term=colon+cancer+exercise+and+metformin&rank=1**](http://www.clinicaltrials.gov/ct2/show/NCT01340300?term=colon+cancer+exercise+and+metformin&rank=1)**.**

**83. Kuiper JG, Phipps AI, Neuhouser ML, Chlebowski RT, Thomson CA, Irwin ML, et al. Recreational physical activity, body mass index, and survival in women with colorectal cancer. Cancer Causes Control 2012.**

**84. Quadrilatero J, Hoffman-Goetz L Physical activity and colon cancer. A systematic review of potential mechanisms. J Sports Med Phys Fitness 2003; 43:121-138.**

**85. Rock CL, Doyle C, Demark-Wahnefried W, Meyerhardt J, Courneya KS, Schwartz AL, et al. Nutrition and physical activity guidelines for cancer survivors. CA Cancer J Clin 2012.**

**86. Schmitz KH, Courneya KS, Matthews C, Demark-Wahnefried W, Galvao DA, Pinto BM, et al. American college of sports medicine roundtable on exercise guidelines for cancer survivors. Med Sci Sports Exerc 2010; 42:1409-1426.**

**87. Brown JC, Huedo-Medina TB, Pescatello LS, Pescatello SM, Ferrer RA, Johnson BT Efficacy of exercise interventions in modulating cancer-related fatigue among adult cancer survivors: a meta-analysis. Cancer Epidemiol Biomarkers Prev 2011; 20:123-133.**

**88. Brown JC, Huedo-Medina TB, Pescatello LS, Ryan SM, Pescatello SM, Moker E, et al. The efficacy of exercise in reducing depressive symptoms among cancer survivors: a meta-analysis. PLoS One 2012; 7:e30955.**

**89. Galvao DA, Newton RU Review of exercise intervention studies in cancer patients. J Clin Oncol 2005; 23:899-909.**

**90. Lee IM Dose-response relation between physical activity and fitness: even a little is good; more is better. JAMA 2007; 297:2137-2139.**

**91. Schmitz KH, Ahmed RL, Troxel A, Cheville A, Smith R, Lewis-Grant L, et al. Weight lifting in women with breast-cancer-related lymphedema. N Engl J Med 2009; 361:664-673.**

**92. Schmitz KH, Ahmed RL, Troxel AB, Cheville A, Lewis-Grant L, Smith R, et al. Weight lifting for women at risk for breast cancer-related lymphedema: a randomized trial. JAMA 2010; 304:2699-2705.**

**93. Brown JC, Troxel AB, Schmitz KH Safety of Weight-Lifting among Women with or At-Risk for Breast-Cancer-Related Lymphedema: Musculoskeletal Injuries and Healthcare Utilization in a Weight-Lifting Rehabilitation Trial The Oncologist In Press.**

**94. LIFE Study Investigators, Pahor M, Blair SN, Espeland M, Fielding R, Gill TM, et al. Effects of a physical activity intervention on measures of physical performance: Results of the lifestyle interventions and independence for Elders Pilot (LIFE-P) study. J Gerontol A Biol Sci Med Sci 2006; 61:1157-1165.**

**95. Daley AJ, Bowden SJ, Rea DW, Billingham L, Carmicheal AR What advice are oncologists and surgeons in the United Kingdom giving to breast cancer patients about physical activity? Int J Behav Nutr Phys Act 2008; 5:46.**

**96. Giovannucci EL Physical activity as a standard cancer treatment. J Natl Cancer Inst 2012; 104:797-799.**

**97. Jones LW, Courneya KS, Peddle C, Mackey JR Oncologists' opinions towards recommending exercise to patients with cancer: a Canadian national survey. Support Care Cancer 2005; 13:929-937.**

**98. Blankenberg S, Rupprecht HJ, Bickel C, Peetz D, Hafner G, Tiret L, et al. Circulating cell adhesion molecules and death in patients with coronary artery disease. Circulation 2001; 104:1336-1342.**

**99. Adamopoulos S, Parissis J, Kroupis C, Georgiadis M, Karatzas D, Karavolias G, et al. Physical training reduces peripheral markers of inflammation in patients with chronic heart failure. Eur Heart J 2001; 22:791-797.**

**100. Hamdy O, Ledbury S, Mullooly C, Jarema C, Porter S, Ovalle K, et al. Lifestyle modification improves endothelial function in obese subjects with the insulin resistance syndrome. Diabetes Care 2003; 26:2119-2125.**

**101. Leinonen E, Hurt-Camejo E, Wiklund O, Hulten LM, Hiukka A, Taskinen MR Insulin resistance and adiposity correlate with acute-phase reaction and soluble cell adhesion molecules in type 2 diabetes. Atherosclerosis 2003; 166:387-394.**

**102. Tonjes A, Scholz M, Fasshauer M, Kratzsch J, Rassoul F, Stumvoll M, et al. Beneficial effects of a 4-week exercise program on plasma concentrations of adhesion molecules. Diabetes Care 2007; 30:e1.**

**103. Saetre T, Enoksen E, Lyberg T, Stranden E, Jorgensen JJ, Sundhagen JO, et al. Supervised exercise training reduces plasma levels of the endothelial inflammatory markers E-selectin and ICAM-I in patients with peripheral arterial disease. Angiology 2011; 62:301-305.**

**104. Craft LL, Guralnik JM, Ferrucci L, Liu K, Tian L, Criqui MH, et al. Physical activity during daily life and circulating biomarker levels in patients with peripheral arterial disease. Am J Cardiol 2008; 102:1263-1268.**

**105. Kraus WE, Torgan CE, Duscha BD, Norris J, Brown SA, Cobb FR, et al. Studies of a targeted risk reduction intervention through defined exercise (STRRIDE). Med Sci Sports Exerc 2001; 33:1774-1784.**

**106. Slentz CA, Duscha BD, Johnson JL, Ketchum K, Aiken LB, Samsa GP, et al. Effects of the amount of exercise on body weight, body composition, and measures of central obesity: STRRIDE--a randomized controlled study. Arch Intern Med 2004; 164:31-39.**

**107. Slentz CA, Aiken LB, Houmard JA, Bales CW, Johnson JL, Tanner CJ, et al. Inactivity, exercise, and visceral fat. STRRIDE: a randomized, controlled study of exercise intensity and amount. J Appl Physiol 2005; 99:1613-1618.**

**108. Slentz CA, Houmard JA, Kraus WE Exercise, abdominal obesity, skeletal muscle, and metabolic risk: evidence for a dose response. Obesity 2009; 17:S27-S33.**

**109. Dutheil F, Lac G, Lesourd B, Chapier R, Walther G, Vinet A, et al. Different modalities of exercise to reduce visceral fat mass and cardiovascular risk in metabolic syndrome: the RESOLVE* randomized trial. Int J Cardiol 2013; 168:3634-3642.**

**110. Ohkawara K, Tanaka S, Miyachi M, Ishikawa-Takata K, Tabata I A dose–response relation between aerobic exercise and visceral fat reduction: systematic review of clinical trials. Int J Obes 2007; 31:1786-1797.**

**111. Vissers D, Hens W, Taeymans J, Baeyens J, Poortmans J, Van Gaal L The Effect of Exercise on Visceral Adipose Tissue in Overweight Adults: A Systematic Review and Meta-Analysis. PloS one 2013; 8:e56415.**

**112. Friedenreich CM, Neilson HK, Woolcott CG, McTiernan A, Wang Q, Ballard-Barbash R, et al. Changes in insulin resistance indicators, IGFs, and adipokines in a year-long trial of aerobic exercise in postmenopausal women. Endocr Relat Cancer 2011; 18:357-369.**

**113. Heydari M, Freund J, Boutcher S The effect of high-intensity intermittent exercise on body composition of overweight young males. Journal of obesity 2012; 2012.**

**114. Houmard JA, Tanner CJ, Slentz CA, Duscha BD, McCartney JS, Kraus WE Effect of the volume and intensity of exercise training on insulin sensitivity. J Appl Physiol (1985) 2004; 96:101-106.**

**115. Ford ES, Li C, Zhao G, Pearson WS, Tsai J, Churilla JR Sedentary behavior, physical activity, and concentrations of insulin among US adults. Metab Clin Exp 2010; 59:1268-1275.**

**116. Mayer-Davis EJ, D'Agostino R,Jr, Karter AJ, Haffner SM, Rewers MJ, Saad M, et al. Intensity and amount of physical activity in relation to insulin sensitivity: the Insulin Resistance Atherosclerosis Study. JAMA 1998; 279:669-674.**

**117. O'Leary VB, Marchetti CM, Krishnan RK, Stetzer BP, Gonzalez F, Kirwan JP Exercise-induced reversal of insulin resistance in obese elderly is associated with reduced visceral fat. J Appl Physiol 2006; 100:1584-1589.**

**118. R&D Systems Human sICAM-1/CD54 Immunoassay. . Available from:** [**http://www.rndsystems.com/pdf/DCD540.pdf**](http://www.rndsystems.com/pdf/DCD540.pdf)**.**

**119. R&D Systems Human sVCAM-1 Immunoassay. . Available from:** [**http://www.rndsystems.com/pdf/dvc00.pdf**](http://www.rndsystems.com/pdf/dvc00.pdf)**.**

**120. Diamond E, Lee GY, Akhtar NH, Kirby BJ, Giannakakou P, Tagawa ST, et al. Isolation and characterization of circulating tumor cells in prostate cancer. Front Oncol 2012; 2:131.**

**121. Gleghorn JP, Pratt ED, Denning D, Liu H, Bander NH, Tagawa ST, et al. Capture of circulating tumor cells from whole blood of prostate cancer patients using geometrically enhanced differential immunocapture (GEDI) and a prostate-specific antibody. Lab Chip 2010; 10:27-29.**

**122. Bell GI, Pictet RL, Rutter WJ, Cordell B, Tischer E, Goodman HM Sequence of the human insulin gene. Nature 1980; 284:26-32.**

**123. Wallace TM, Levy JC, Matthews DR Use and abuse of HOMA modeling. Diabetes Care 2004; 27:1487-1495.**

**124. Kaul S, Rothney MP, Peters DM, Wacker WK, Davis CE, Shapiro MD, et al. Dual‐Energy X‐Ray Absorptiometry for Quantification of Visceral Fat. Obesity 2012; 20:1313-1318.**

**125. Micklesfield LK, Goedecke JH, Punyanitya M, Wilson KE, Kelly TL Dual‐Energy X‐Ray Performs as Well as Clinical Computed Tomography for the Measurement of Visceral Fat. Obesity 2012; 20:1109-1114.**

**126. Dauer LT, Thornton RH, Hay JL, Balter R, Williamson MJ, St Germain J Fears, feelings, and facts: interactively communicating benefits and risks of medical radiation with patients. AJR Am J Roentgenol 2011; 196:756-761.**

**127. Shephard RJ PAR-Q, Canadian Home Fitness Test and exercise screening alternatives. Sports Med 1988; 5:185-195.**

**128. ATS Committee on Proficiency Standards for Clinical Pulmonary Function Laboratories ATS statement: guidelines for the six-minute walk test. Am J Respir Crit Care Med 2002; 166:111-117.**

**129. Gill TM, DiPietro L, Krumholz HM Role of exercise stress testing and safety monitoring for older persons starting an exercise program. JAMA: the journal of the American Medical Association 2000; 284:342-349.**

**130. Pahor M, Guralnik JM, Ambrosius WT,et al Effect of structured physical activity on prevention of major mobility disability in older adults: The life study randomized clinical trial. JAMA 2014.**

**131. Ingle L, Shelton RJ, Rigby AS, Nabb S, Clark AL, Cleland JG The reproducibility and sensitivity of the 6-min walk test in elderly patients with chronic heart failure. Eur Heart J 2005; 26:1742-1751.**

**132. Bean JF, Kiely DK, Leveille SG, Herman S, Huynh C, Fielding R, et al. The 6-minute walk test in mobility-limited elders: what is being measured? J Gerontol A Biol Sci Med Sci 2002; 57:M751-6.**

**133. Bruce RA, Kusumi F, Hosmer D Maximal oxygen intake and nomographic assessment of functional aerobic impairment in cardiovascular disease. Am Heart J 1973; 85:546-562.**

**134. Foster C, Jackson AS, Pollock ML, Taylor MM, Hare J, Sennett SM, et al. Generalized equations for predicting functional capacity from treadmill performance. Am Heart J 1984; 107:1229-1234.**

**135. Luhrmann PM, Herbert BM, Gaster C, Neuhauser-Berthold M Validation of a self-administered 3-day estimated dietary record for use in the elderly. Eur J Nutr 1999; 38:235-240.**

**136. John D, Tyo B, Bassett DR Comparison of four ActiGraph accelerometers during walking and running. Med Sci Sports Exerc 2010; 42:368-374.**

**137. Treuth MS, Schmitz K, Catellier DJ, McMurray RG, Murray DM, Almeida MJ, et al. Defining accelerometer thresholds for activity intensities in adolescent girls. Med Sci Sports Exerc 2004; 36:1259-1266.**

**138. Van Coevering P, Harnack L, Schmitz K, Fulton JE, Galuska DA, Gao S Feasibility of using accelerometers to measure physical activity in young adolescents. Med Sci Sports Exerc 2005; 37:867-871.**

**139. Sirard JR, Forsyth A, Oakes JM, Schmitz KH Accelerometer test-retest reliability by data processing algorithms: results from the Twin Cities Walking Study. J Phys Act Health 2011; 8:668-674.**

**140. Stein AD, Lederman RI, Shea S The Behavioral Risk Factor Surveillance System questionnaire: its reliability in a statewide sample. Am J Public Health 1993; 83:1768-1772.**

**141. Gama H, Correia S, Lunet N Effect of questionnaire structure on recall of drug utilization in a population of university students. BMC Med Res Methodol 2009; 9:45.**

**142. Ward WL, Hahn EA, Mo F, Hernandez L, Tulsky DS, Cella D Reliability and validity of the Functional Assessment of Cancer Therapy-Colorectal (FACT-C) quality of life instrument. Qual Life Res 1999; 8:181-195.**

**143. Ware JE,Jr, Sherbourne CD The MOS 36-item short-form health survey (SF-36). I. Conceptual framework and item selection. Med Care 1992; 30:473-483.**

**144. Harrington CB, Hansen JA, Moskowitz M, Todd BL, Feuerstein M It's not over when it's over: long-term symptoms in cancer survivors--a systematic review. Int J Psychiatry Med 2010; 40:163-181.**

**145. Committee on Cancer Survivorship: Institute of Medicine and national Research Board From Cancer Patient to Cancer Survivor: Lost in Transition. 2006.**

**146. Cleeland CS, Ryan KM Pain assessment: global use of the Brief Pain Inventory. Ann Acad Med Singapore 1994; 23:129-138.**

**147. Buysse DJ, Reynolds CF,3rd, Monk TH, Berman SR, Kupfer DJ The Pittsburgh Sleep Quality Index: a new instrument for psychiatric practice and research. Psychiatry Res 1989; 28:193-213.**

**148. Haddock MG, Sloan JA, Bollinger JW, Soori G, Steen PD, Martenson JA, et al. Patient assessment of bowel function during and after pelvic radiotherapy: results of a prospective phase III North Central Cancer Treatment Group clinical trial. J Clin Oncol 2007; 25:1255-1259.**

**149. Mendoza TR, Wang XS, Cleeland CS, Morrissey M, Johnson BA, Wendt JK, et al. The rapid assessment of fatigue severity in cancer patients: Use of the brief fatigue inventory. Cancer 1999; 85:1186-1196.**

**150. Galer BS, Jensen MP Development and preliminary validation of a pain measure specific to neuropathic pain: the Neuropathic Pain Scale. Neurology 1997; 48:332-338.**

**151. Warren M, Schmitz KH Safety of strength training in premenopausal women: musculoskeletal injuries from a two-year randomized trial. Am J Health Promot 2009; 23:309-314.**

**152. Thompson, WR., Gordon, NF., Pescatello LS. ACSM's Guidelines for Exercise Testing and Prescription. 2010.**

**153. Schmitz KH ACSM's Guidelines for Exercise Testing and Prescription 9th. ed. Chapter 10 - Cancer. In: Anonymous , 2013.**

**154. Weinberg RS Goal setting and performance in sport and exercise settings: a synthesis and critique. Med Sci Sports Exerc 1994; 26:469-477.**

**155. Hochberg Y A sharper Bonferroni procedure for multiple tests of significance. Biometrika 1988; 75:800-802.**
